# M^6^A Demethyltransferase FTO Attenuates Meniscus Degeneration and Osteoarthritis via Orchestrating Autophagy and Energetic Metabolism

**DOI:** 10.1002/advs.202412379

**Published:** 2025-01-13

**Authors:** Zongrui Jiang, Chengyun Zhang, Ruonan Liu, Zijing Zhu, Dianbo Long, Xingzhao Wen, Zhijian Yang, Dong Jiang, Guping Mao, Weiming Liao, Zhiqi Zhang

**Affiliations:** ^1^ Department of Joint Surgery The First Affiliated Hospital of Sun Yat‐sen University Guangzhou Guangdong 510080 China; ^2^ Department of Sport Medicine The First Affiliated Hospital of Sun Yat‐sen University Guangzhou Guangdong 510080 China; ^3^ Guangdong Provincial Key Laboratory of Orthopedics and Traumatology Guangzhou Guangdong 510080 China

**Keywords:** ATG16L1, autophagy, energetic metabolism, FTO, meniscus degeneration, N6‐methyladenosine

## Abstract

Impaired autophagy is reported to promote osteoarthritis (OA). However, the mechanism by which autophagy in regulating meniscus degeneration and OA remains unclear. Here, unconvered aberrant energetic metabolism pattern in meniscus cells with OA is uncovered first, which results in lower adenosine triphosphate (ATP) production. And these phenomena are induced by impaired autophagy in meniscus cells with OA. It is further revealed that the suppression of m^6^A demethylase fat mass and obesity‐associated protein (FTO) inhibits autophagy and causing lower ATP production by reducing oxidative phosphorylation. Specific deletion of FTO in meniscus cells by generating FTO^flox/flox^; COL1A1‐Cre^ERT2^ (FTO^cko^) mice impair autophagy and promote meniscus degeneration and OA, while intra‐articular injection of adeno‐associated virus of FTO (AAV‐FTO) restores autophagy and alleviates meniscus degeneration and OA. Mechanistically, FTO regulates the mRNA stability of ATG16L1 by targeting the m^6^A methylation sites on ATG16L1 in a YTHDF2‐dependent manner, thereby inhibiting the formation of autophagosomes and causing an imbalance in energetic metabolism. Intra‐articular injection of AAV‐FTO reverses the catabolic phenotype of meniscus degeneration and OA in FTO^cko^ mice. In summary, these findings reveal FTO orchestrates autophagy and energetic metabolism by regulating ATG16L1 in a m^6^A‐dependent manner. Therefore, targeting FTO might be a potential therapeutic strategy for meniscus degeneration and early‐stage OA.

## Graphical abstract

Autophagy and energy metabolism are crucial to cellular homeostasis. Zhang et al. have unveiled that Fat Mass And Obesity‐Associated Protein (FTO) orchestrates autophagy and energetic metabolism in meniscus cells via ATG16L1 in a m^6^A‐dependent manner. FTO overexpression restores autophagy and energetic metabolism in both destabilization of the medial meniscus‐induced osteoarthritis models and FTO conditional knockout mice in meniscus cells (FTO^cko^). These results implicate FTO as a potential therapeutic target for meniscus degeneration.

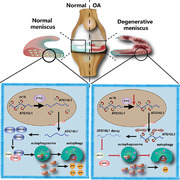

## 1 Introduction

Menisci, with its specific semi‐lunar anatomical structure located between distal femur and proximal tibia, is one of the most essential components in the knee joint. Meniscus provides cushions, stability, and shock absorptance to cartilage between femur and tibia and hence maintains the biomechanical homeostasis of knee joint.^[^ ^1^ ^]^ Based on the distribution of blood vessels and nerves between inner region and outer region of meniscus, it could be divided into red zone (region with blood vessels and nerves) and white zone (region without blood vessels and nerves).^[^ ^2^ ^]^ Meniscus tears, especially within white zone are often irreparable due to absence of blood supply.^[^ ^3^ ^]^ Evidence have already suggested that patients with magnetic resonance imaging (MRI)‐diagnosed degenerative meniscus tears show higher risk of early‐stage osteoarthritis (OA), a common degenerative disease worldwide.^[^ ^3^, ^4^, ^5^, ^6^ ^]^ However, the specific mechanism of how meniscus degeneration induces early‐stage OA remains elusive. Our previous studies reported that degenerative meniscus with OA released meniscus cell lysate and promotes the secretion of inflammatory cytokines of fibroblast‐like synoviocytes (FLS).^[^ ^5^ ^]^ Also, research have shown that meniscus tissue repair with transcription factor Mohawk and Transformative growth factor β3 could also alleviate OA.^[^ ^7^ ^]^ These studies have stressed out the importance of meniscus in the pathogenesis of OA. Since arthroscopic surgery has been reported to have limited therapeutic effects on meniscus degeneration,^[^ ^8^ ^]^ it is of great significance to understand the underlying mechanism and discover potential targets for treating meniscus degeneration to prevent OA.

Our former study and related research suggest that meniscus degeneration is tightly related with abnormal cellular homeostasis.^[^ ^9^ ^]^ Among multiple factors that might affect cellular homeostasis, aberrant bioenergetic metabolism is one of the most important elements that cannot be negligible.^[^ ^10^, ^11^ ^]^ In our study, we found dramatically greater enrichment of metabolites derived from glycolysis in meniscus cells isolated from OA patients. Metabolic reprogramming, such as aerobic glycolysis, is a relatively contradictory metabolic process in which glucose is mainly catalyzed into lactate in an aerobic microenvironment.^[^ ^12^ ^]^ This process is also known as metabolic adaptation for robust tumor proliferation and expansion.^[^ ^12^, ^13^, ^14^ ^]^ However, sustained aerobic glycolysis causes dysfunctional metabolism in chondrocytes, which is believed to be involved in the OA process.^[^ ^15^, ^16^ ^]^ Evidence also indicates that dysfunctional metabolism is closely related to impaired autophagy.^[^ ^17^ ^]^ As one of the most advanced cleaning systems in cells, autophagy is reported to maintain the balance of energy metabolism via selective autophagic lysis of aberrant organelles or by providing various substrates to fuel metabolism.^[^ ^18^ ^]^ Interestingly, autophagy also serves as the primary mechanism for the survival of chondrocytes,^[^ ^19^ ^]^ and dysregulated autophagy in chondrocytes, FLSs, and meniscus cells has been shown to promote OA progression.^[^ ^20^, ^21^, ^22^ ^]^ However, the relationship between impaired autophagy and aberrant energy metabolism in patients with meniscus degeneration the underlying mechanism remain uncertain.

Epigenetic regulation is necessary for maintaining cell viability and growth.^[^ ^23^ ^]^ It affects almost every intracellular biological process, including autophagy and metabolism.^[^ ^24^, ^25^ ^]^ It has recently been reported that N6‐methyladenosine (m^6^A) is one of the most common epigenetic modifications of messenger RNA (mRNA) and it regulates the stability, slicing, transport, and translation of certain mRNAs.^[^ ^26^ ^]^ M^6^A methylation has been reported to have important regulating role in autophagy and other biological process. For instance, m^6^A methylation has been studied to sustain the mRNA stability of autophagy‐related genes 5 (ATG5) and ATG7, which plays a critical role in the formation of autophagosome in a m^6^A‐dependent manner and thereby maintain the interplay between autophagy and adipogenesis.^[^ ^24^ ^]^ Aside from typical ATGs, it has also been found that m^6^A methylation regulates the mRNA stability of P62/SQSTM1, a well‐known substrate in autophagy, hence affecting the selective lysosomal degradation system.^[^ ^27^ ^]^ These studies have all suggested that m^6^A methylation are essentially important in regulating autophagy. Therefore, we proposed that m^6^A methylation might regulate energetic metabolism via autophagy flux.

In our study, we revealed that impaired autophagy resulted in dysfunctional bioenergetic metabolism and decreased ATP production. We identified and validated that the m^6^A “eraser” FTO maintained autophagy and energetic metabolism by modulating the stability of the ATG16L1 mRNA transcript in an m^6^A‐dependent manner. Thus, our study unveiled the role and mechanism of m^6^A methylation in orchestrating the interplay of autophagy and energetic metabolism of meniscus and offered a brand‐new insight and cognition in meniscus degeneration and OA. This finding also implicated that FTO might be a potential target for treating meniscus degeneration and OA.

## 2 Results

### 2.1 Abnormal Bioenergetic Metabolism in Meniscus Cells Relates with Meniscus Degeneration in OA

**Figure 1:**
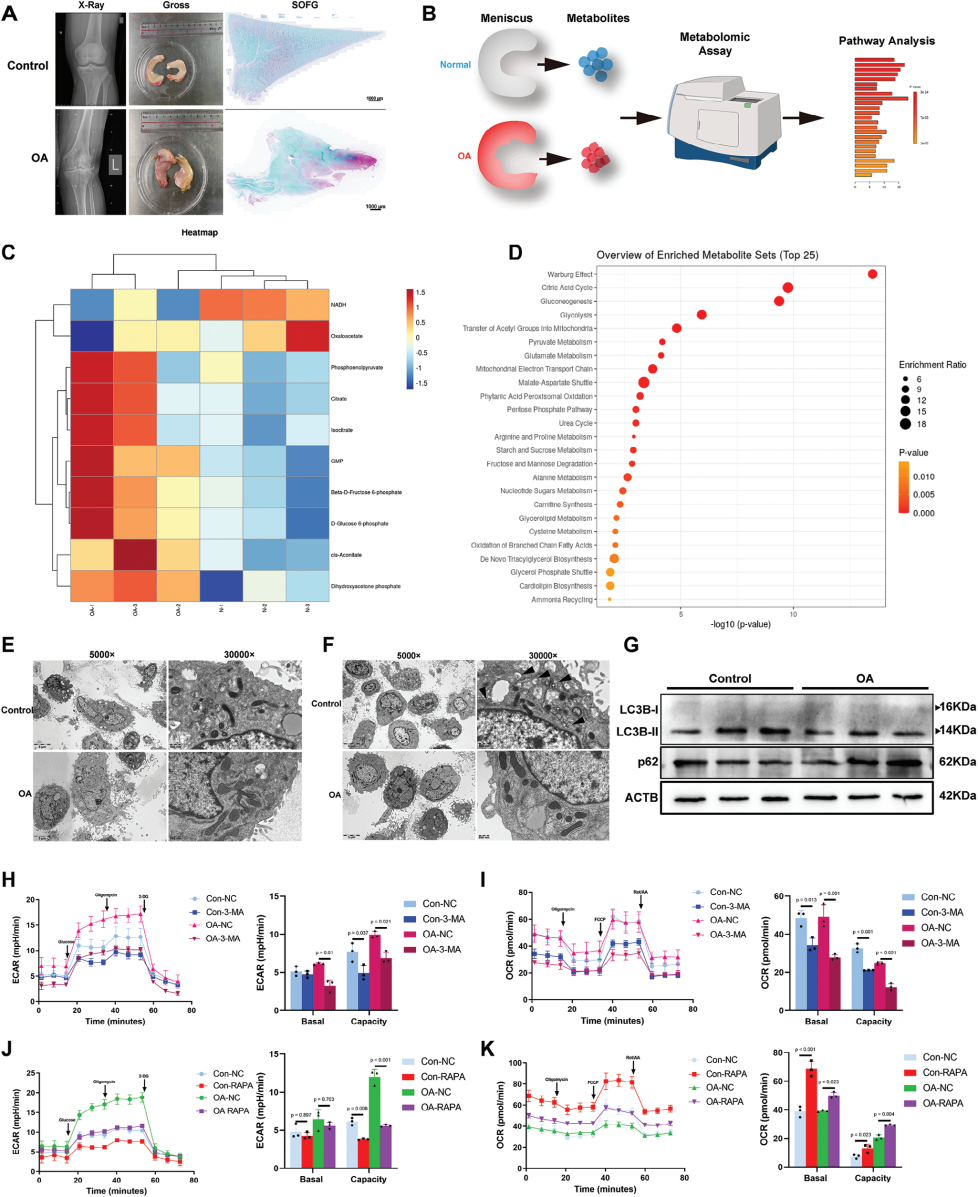
Impaired autophagy promotes dysfunctional bioenergetic metabolism in meniscus degeneration. A) X‐ray image of the osteoarthritic (OA) knee and normal control knee without OA (Control) and gross morphology and Safranin‐O/fast green (SOFG) staining of the meniscus dissected from patients with and without OA. B) Schematic diagram of the effect of targeted metabolomics on energy metabolism. C) Heatmap of differentially enriched metabolites related to energy metabolism. D) Enrichment analysis of metabolites related to metabolism. E) Classical transmissional electron microscopy images showing the ultrastructure of dysfunctional mitochondria in meniscus cells from patients with OA. Scale bars, 5 µm (left) and 500 nm (right). F) Representative images showing the decrease in the ultrastructure of autophagosomes in meniscus cells isolated from OA patients. The black arrows indicate autophagosomes. Scale bar, 500 nm. G) Western blot (WB) analysis of LC3B and p62 in meniscus cells from patients with OA and patients without OA (control). *n* = 3 per group. H–K) ECAR and OCR analysis of meniscus cells from the control group and OA group treated with or without the autophagy inhibitor 3‐MA and the autophagy stimulator rapamycin. *n* = 3 per group. H–K) All the data are presented as the mean ± SEM (standard error of mean). One‐way analysis of variance (ANOVA) followed with Turky’ HSD test was used for statistical analysis. *P*‐values are presented in each bar plot.

To concisely identify cellular energetic metabolism during meniscus degeneration, we dissected meniscus tissues from patients with or without OA and performed targeted metabolomics for bioenergetic metabolism (**Figure** **1**A,B and Table S1, Supporting Information). Several metabolites, such as alpha‐ketoglutarate and nicotinamide adenine dinucleotide (NADH), were highly enriched in the meniscus without OA, while necessary glycolytic metabolites, such as phosphoenolpyruvate, beta−D−fructose 6−phosphate, D−glucose 6−phosphate, and dihydroxyacetone phosphate, were mainly distributed in the meniscus isolated from OA patients (Figure 1C). Pathway enrichment analysis was performed using all Kyoto Encyclopedia of Genes and Genomes metabolic pathway classes, and the results indicated that the Warburg effect was significantly enriched in the degenerated meniscus of patients with OA (Figure 1D).

We compared the glycolytic rates and mitochondrial respiration in meniscus cells with and without OA. Seahorse real‐time cell metabolic analysis revealed that the bioenergetic metabolic status switched from mitochondrial oxidative phosphorylation (OXPHO) to glycolysis (Figure S1A,B, Supporting Information). ATP production in meniscus cells from OA patients was significantly lower than that in meniscus cells from patients without OA (Figure S1C, Supporting Information). Based on these results, we further evaluated the mitochondrial membrane potential (Δψm) in meniscus cells. Confocal microscopy and flow cytometry of mitochondrial membrane‐potential dye (JC‐1) fluorescence showed that the Δψm was significantly lower in meniscus cells from OA patients (Figure S1D, Supporting Information). Mitochondrial reactive oxygen species (mROS) and cytosolic ROS accumulated in meniscus cells with OA (Figure S1E,F, Supporting Information). Transmission electron microscopy (TEM) revealed that the mitochondria in the meniscus cells from the OA patients were noticeably swollen (Figure 1E and Figure S1G, Supporting Information). These results indicated abnormal bioenergetic metabolism in meniscus degeneration.

### 2.2 Impaired Autophagy Contributes to Dysregulated Energy Metabolism in Meniscus Degeneration

We further investigated how aberrant bioenergetic metabolic reprogramming occurs in meniscus degeneration. It has been reported that autophagy is strongly related to metabolic disorders and dysregulated energy metabolism.^[^ ^28^ ^]^ Dysregulated autophagy also contributes to cartilage degeneration during OA.^[^ ^29^ ^]^ Surprisingly, along with swollen and fragmented mitochondria, fewer autophagosomes were detected in meniscus cells from patients with OA (Figure 1F and Figure S1H, Supporting Information). Western blot (WB) analysis revealed that the levels of LC3B‐II (a classic biomarker of autophagosomes) were significantly decreased in meniscus cells from OA patients compared with that from patients without OA, while the levels of p62 (a protein regulating autophagy of abnormal organelles and aggregates) was upregulated in meniscus cells with OA (Figure 1G and Figure S2A, Supporting Information). Additionally, immunofluorescence (IF) revealed increased levels of p62 and decreased LC3B expression in meniscus cells from OA patients (Figure S2B, Supporting Information). Moreover, we verified whether autophagy was also dysregulated in aging and destabilization of the medial meniscus (DMM)‐induced OA mice. First, the Osteoarthritis Research Society International (OARSI) score^[^ ^30^ ^]^ and meniscus score^[^ ^31^ ^]^ confirmed that cartilage and meniscus degeneration were aggravated during aging (Figure S2C, Supporting Information). The expression of LC3B was also reduced in the anterior meniscus of aging mice (Figure S2D, Supporting Information). Moreover, mice were subjected to DMM surgery and the knees were collected at 2, 4, 6, and 8 weeks after DMM surgery. The OARSI score and meniscus score based on Safranin O/Fast green (SOFG) staining showed that meniscus injury and degeneration preceded cartilage damage and aggravated with time until 6 weeks after DMM surgery, suggesting that meniscus degeneration is closely related to OA (Figure S2E, Supporting Information). Immunofluorescence of LC3B revealed that the expression of LC3B decreased following DMM‐induced OA in mice (Figure S2F, Supporting Information). These results indicated that impaired autophagy occurs in meniscus degeneration. To validate the hypothesis that impaired autophagy induces dysfunctional energy metabolism, we performed real‐time metabolic analysis with a Seahorse system. The autophagy inhibitor 3‐methyladenine (3‐MA)^[^ ^32^ ^]^ significantly inhibited the basic and compensatory extracellular acidification rates (ECARs) in meniscus cells isolated from patients with and without OA (Figure 1H). Mitochondrial stress tests revealed decreased basic and compensatory respiratory capacity in mitochondria (Figure 1I). On the other hand, rapamycin (RAPA), an autophagy activator,^[^ ^33^ ^]^ alleviated the increase in glycolysis (Figure 1J) and rescued oxidative phosphorylation and ATP production in meniscus cells (Figure 1K). Overall, impaired autophagy in degenerative meniscus cells promotes bioenergetic metabolic reprogramming and decreases ATP production during OA.

### 2.3 The m^6^A Demethyltransferase FTO Rescues Autophagy and Alleviates Aberrant Metabolic Reprogramming in Meniscus Cells

**Figure 2:**
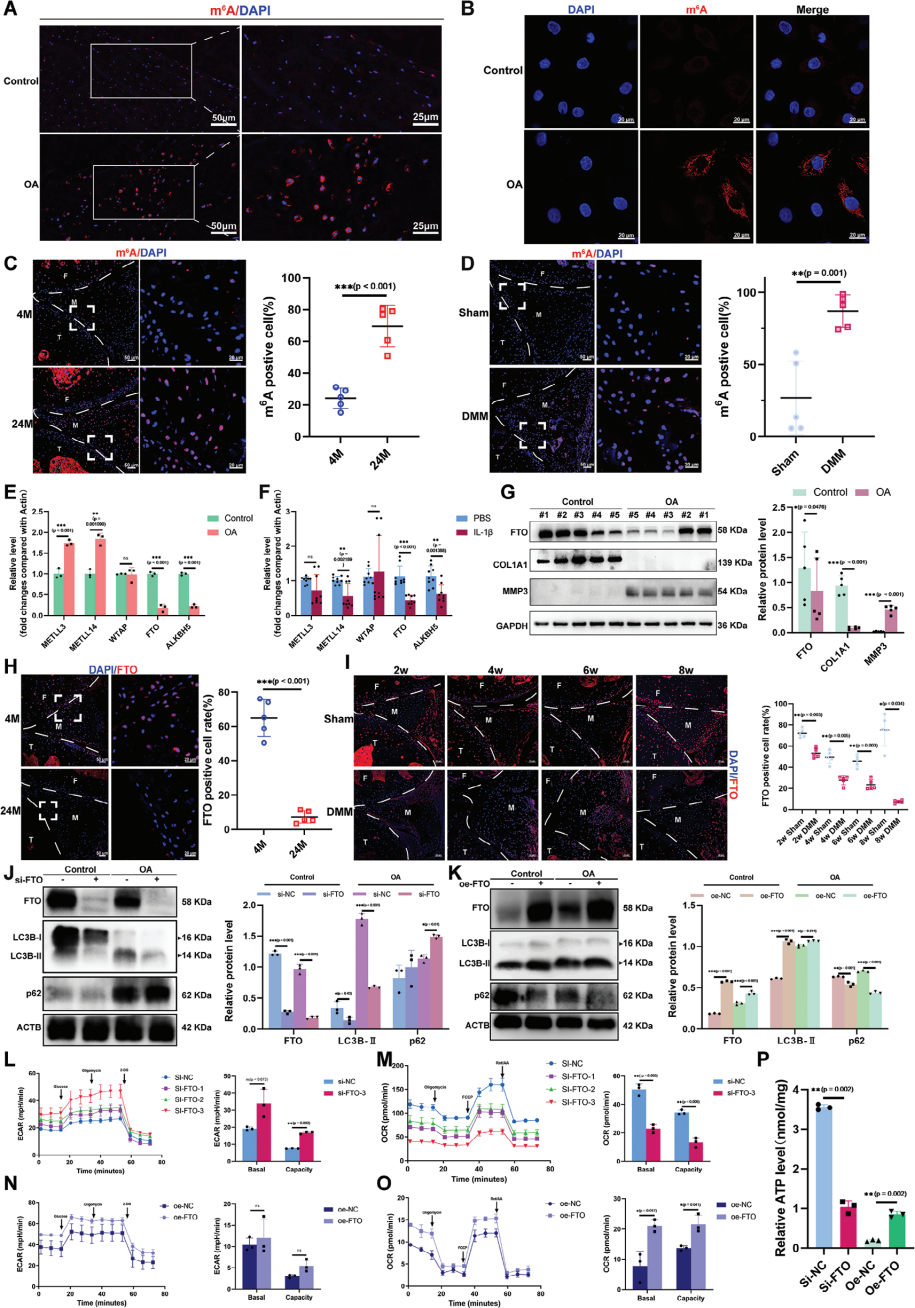
Enhanced m^6^A methylation caused by downregulation of FTO contributes to impaired autophagy and metabolic reprogramming in the degenerating meniscus. A) Representative IHC image of m^6^A (red) in meniscus tissues from patients with OA and patients without OA (control). Scale bars, 100 µm (left) and 50 µm (right). B) Representative IF staining of m^6^A in meniscus cells from the OA group and control group. Scale bar, 20 µm. C) IF staining and quantification of m^6^A in the meniscus of knee joints from 4‐month‐old mice and 24‐month‐old mice. *n* = 5 per group. Scale bars, 50 µm (left) and 20 µm (right). D) IF staining and quantification of m^6^A‐positive meniscus cells in osteoarthritic knee joints from mice that underwent DMM surgery for 8 weeks. *n* = 5 per group. Scale bars, 50 µm (left) and 20 µm (right). E) qRT‒PCR analysis of several m^6^A methyltransferases (METLL3, METLL14, and WTAP) and demethyltransferases (FTO and ALKBH5) in meniscus cells from OA patients and normal patients. *n* = 3 per group. F) qRT‒PCR analysis of the levels of several m^6^A methyltransferases (METLL3, METLL14, and WTAP) and demethyltransferases (FTO and ALKBH5) in meniscus cells treated with or without IL‐1β. *n* = 9 per group. G) WB analysis and evaluation of the protein levels of FTO, COL1A1, MMP3, and glyceraldehyde‐3‐phosphate dehydrogenase (GAPDH). *n* = 5 per group. H) IF staining and quantification of FTO in the meniscus tissues of 4‐month‐old mice and 24‐month‐old mice. *n* = 5 per group. I) Representative IF staining and quantification of FTO in the meniscus tissue from DMM patients 2, 4, 6, and 8 weeks after surgery. *n* = 5 per group. J,K) The protein levels and quantification of FTO, LC3B, and p62 in meniscus cells with or without small interfering RNA (si‐FTO)‐mediated knockdown of FTO and plasmids overexpressing FTO (oe‐FTO). L,M) ECAR and OCR analysis of meniscus cells with and without transfection of three different phenotypes of si‐FTO. *n* = 3 per group. N,O) The ECAR and OCR of meniscus cells with and without oe‐FTO transfection. *n* = 3 per group. P) ATP levels in meniscus cells treated with or without si‐FTO or oe‐FTO. *n* = 3 per group. All quantification data are presented as E,F,G,J–P) the mean ± SEM and C,D,H,I) median 25th–75th percentiles. G) Paired Student's *t*‐test, C–H,L–O) unpaired Student's *t*‐test and I–K,P) one‐way ANOVA followed with Turky’ HSD test were used for statistical analysis and *P*‐values are presented in each bar plot.

Recent studies have stressed the importance of m^6^A methylation of messenger RNA (mRNA) in epigenetic and posttranscriptional regulation.^[^ ^34^ ^]^ Autophagy and metabolism are also regulated by m^6^A methylation in tumorigenesis, inflammation, and other cellular processes.^[^ ^35^ ^]^ Therefore, we sought to verify whether m^6^A methylation is correlated with meniscal degeneration and OA. Immunofluorescence staining of m^6^6A suggested that m^6^A levels were significantly higher in the meniscus with OA (**Figure** **2**A,B). Consistently, the m^6^A levels were also greater in the menisci of 24‐month‐old aging mice and DMM‐induced OA mice (Figure 2C,D). Dot blot assays also showed that the intensity of m^6^A was greater in the meniscus of the OA and interleukin‐1β (IL‐1β‐induced OA models (Figure S3A,B, Supporting Information). Next, we examined the expression of several m^6^A methyltransferases (METLL3, METLL14, and WTAP) and demethyltransferases (FTO) and ALKBH5) in meniscus cells with and without OA. The quantitative real time‐polymerse chain reaction (qRT‒PCR) results revealed that among these five m^6^A‐related regulators, FTO and ALKBH5 were the most differentially expressed and were significantly downregulated in meniscus cells from OA patients (Figure 2E). IL‐1β stimulation of meniscus cells also significantly downregulated the expression of FTO in meniscus cells (Figure 2F). The protein levels of FTO were also markedly decreased in meniscus cells from OA patients (Figure 2G). Immunostaining also showed that the expression of FTO was downregulated in the meniscus with OA both in vivo and in vitro (Figure S3C,D, Supporting Information). Furthermore, we confirmed the expression pattern of FTO in aging mice (Figure 2H). FTO was downregulated in a time‐dependent manner in DMM‐induced OA mice (Figure 2I). To investigate whether FTO contributes to meniscus degeneration via the regulation of autophagy and glycolysis, we silenced FTO using small interfering RNA (siRNA) and validated the effect of siRNA. qRT‒PCR showed that silencing FTO promoted the expression of matrix metalloproteinases (MMPs), MMP1 and MMP3, while the main extracellular matrix components COL1A1 and type II collagen (COL2A1) were significantly donwregulated (Figure S3E, Supporting Information). Silencing FTO reduced the expression of LC3B‐II and increased the levels of p62 (Figure 2J). In contrast, plasmids encoding FTO (oe‐FTO) were constructed and transfected into meniscus cells, which exhibited decreased expression of matrix metalloproteinases and increased expression of COL1A1 and COL2A1 with or without IL‐1β stimulation (Figure S3F,G, Supporting Information). Overexpressing FTO upregulated the expression of LC3B‐II and inhibited the expression of p62 (Figure 2K). With respect to metabolic reprogramming, silencing FTO promoted glycolysis and abrogated OXPHO (Figure 2L,M). In contrast, transfection of plasmids encoding FTO decreased upregulated glycolysis rates and restored mitochondrial respiration (Figure 2N,O). Moreover, ATP production was lower in meniscus cells after silencing FTO, and overexpressing FTO restored ATP production (Figure 2P). Both results suggested that FTO protected the meniscus from degeneration by modifying autophagy and metabolism.

### 2.4 FTO Overexpression Alleviates Meniscus Degeneration in DMM‐Induced OA Mice

**Figure 3:**
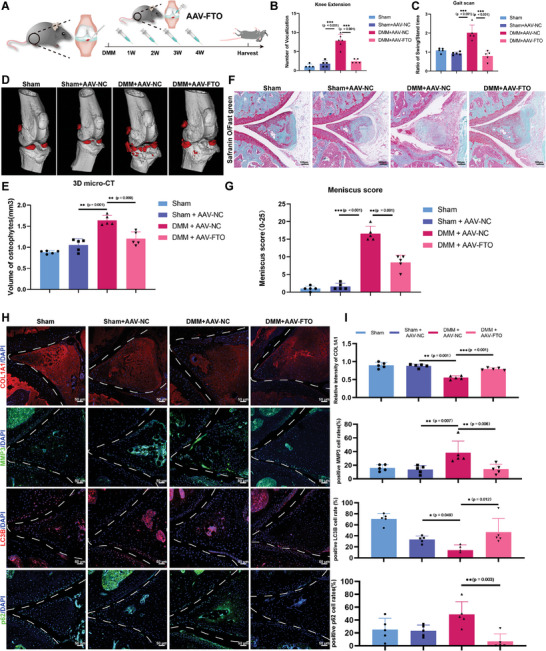
Overexpression of FTO in meniscus cells alleviates DMM‐induced OA in mice. A) Schematic illustration of destabilization of the medial meniscus (DMM) and the duration of intra‐articular injection of adeno‐associated virus overexpressing FTO (AAV‐FTO) at 1, 2, 3, and 4 weeks after DMM surgery. B) Quantification of the number of vocalizations in the knee extension test used for the evaluation of knee pain. *n* = 5 per group. C) The swing/stand time ratio of the rear limb in the Sham, Sham + AAV‐NC, DMM + AAV‐NC, and DMM + AAV‐FTO groups determined using Gaitscan. *n* = 5 per group. D) Representative 3D micro‐CT reconstruction scanning and identification of osteophytes (red) in the Sham, Sham + AAV‐NC, DMM + AAV‐NC, and DMM + AAV‐FTO groups. E) Quantification of the volume of osteophytes (mm^3^). *n* = 5 per group. F) Representative SOFG staining of the meniscus in the knee joints of the sham, sham + AAV‐NC, DMM + AAV‐NC, and DMM + AAV‐FTO groups. G) Quantification of the meniscus score based on SOFG staining in the knee joints of the sham, sham + AAV‐NC, DMM + AAV‐NC, and DMM + AAV‐FTO groups. *n* = 5 per group. H) Representative IF staining of COL1A1 (red), MMP3 (green), LC3B (red), and p62 (green). I) Quantification of the intensity of COL1A1 and the percentages of MMP3‐, LC3B‐, and p62‐positive cells in the anterior meniscus in the knee joints of the Sham, Sham + AAV‐NC, DMM + AAV‐NC, and DMM + AAV‐FTO groups. *n* = 5 per group. B,C,E,G,I) All quantification data are presented as the mean ± SEM. One‐way ANOVA followed with Turky’ HSD test was used for statistical analysis and *p*‐values are presented in each bar plot.

To test whether FTO overexpression in mice could alleviate OA, an adeno‐associated virus containing FTO (AAV‐FTO) was constructed and injected into DMM‐induced OA mice (**Figure** **3**A). The knee extension test revealed severe knee pain, and after DMM surgery, injecting AAV‐FTO further reversed the discomfort caused by DMM (Figure 3B). Gait analysis also revealed a longer latency in the DMM group but a shorter latency in the knee treated with AAV‐FTO (Figure 3C). More osteophytes were detected in the DMM group by micro‐computed tomography (micro‐CT), while overexpressing FTO decreased the number of osteophytes in the mice (Figure 3D,E). The evaluation of the meniscus score based on SOFG staining revealed that meniscus degeneration was mitigated after AAV‐FTO treatment in the DMM model (Figure 3F,G), as was the intensity of type 1 collagen and the ratio of MMP3‐positive cells (Figure 3H,I). FTO overexpression after DMM also increased the level of LC3B and downregulated the expression of p62 (Figure 3H,I). SOFG staining revealed that proteoglycan loss in the cartilage of the DMM group was alleviated by AAV‐FTO injection (Figure S4A,B, Supporting Information). The ratio of aggrecan (ACAN) was elevated by AAV‐FTO with DMM surgery, while the ratio of MMP13‐positive cells was decreased (Figure S4C,D, Supporting Information). Interestingly, hematoxylin‐eosin (HE) staining and synovitis scores were not significantly different between DMM‐induced OA model mice with and without FTO overexpression (Figure S4E,F, Supporting Information). Overall, FTO overexpression in DMM‐induced OA mice had therapeutic effects on cartilage destruction and meniscus degeneration.

### 2.5 FTO Deficiency in Meniscus Cells Accelerates Autophagy‐Related Metabolic Reprogramming and Exacerbates OA Development in Mice

**Figure 4:**
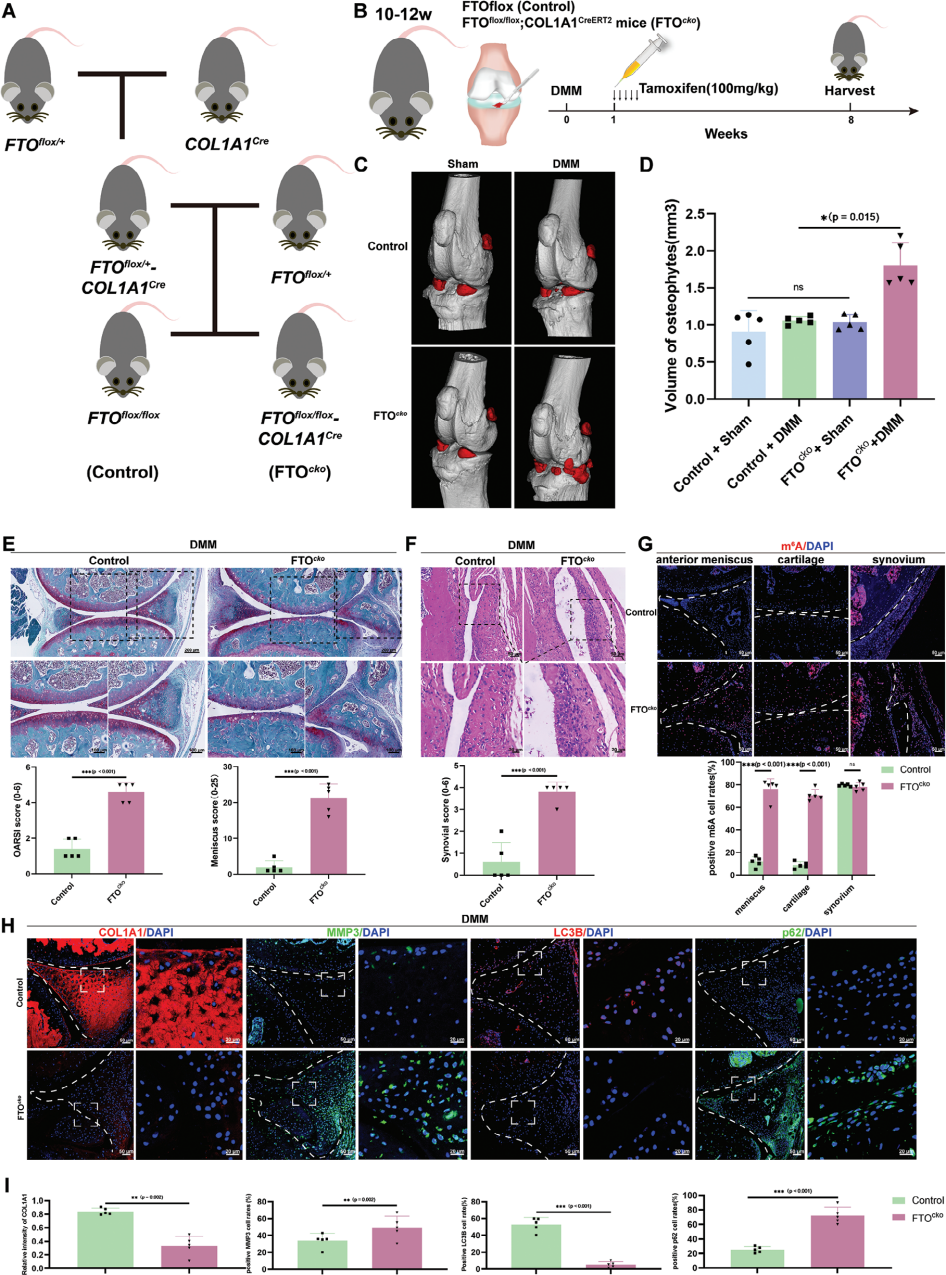
Specific deletion of FTO in meniscus cells exacerbates meniscal degeneration and OA. A) Schematic flowchart of the generation of FTO^flox/flox^; COL1A1‐CreER^T2^ (FTO^cko^) and FTO^flox/flox^ (Control) mice. B) Schematic diagram of the timepoints of intraperitoneal injection of tamoxifen (100 mg kg^−1^) and DMM surgery in the control group and FTO^cko^ group. C) Representative micro‐CT reconstruction and identification of osteophytes (red) in FTO^cko^ and control mice with and without DMM surgery. D) Quantification and analysis of the volume of osteophytes. *n* = 5 per group. E) Representative SOFG of cartilage and meniscus in the control group and FTO^cko^ group with DMM surgery (upper), along with the evaluation of cartilage degeneration and meniscus injury based on the OARSI score and meniscus score, respectively (lower). *n* = 5 per group. The representative HE and SOFG staining of the knee joint of control and FTO^cko^ mice. F) Hematoxylin and eosin (HE) staining of the synovium in the control group and FTO^cko^ group subjected to DMM surgery (upper) and quantification of the synovium score. *n* = 5 per group. G) IF staining of m^6^A (red) in the meniscus, cartilage, and synovium from the knee joints in the control group and FTO^cko^ group, along with the quantification and statistical analysis of the ratio of positive cells in knee tissues. *n* = 5 per group. H,I) Representative IF staining of COL1A1 (red), MMP3 (green), LC3B (red), and p62 (green) and quantification of the intensity of COL1A1 and the percentages of MMP3‐, LC3B‐, and p62‐positive cells in control and FTO^cko^ mice subjected to DMM surgery. Scale bar = 50 µm. D–G,I) All quantification data are presented as the mean ± SEM. E–I) Unpaired Student's *t*‐test and D) one‐way ANOVA followed with Turky’ HSD test was used for statistical analysis and *p*‐values are presented in each bar plot. *p*‐values are presented in each bar plot.

To concisely identify the specific function of FTO in meniscus degeneration and OA progression, we generated FTO^flox/flox^ (Control) and FTO^flox/flox^; COL1A1‐CreER^T2^ mice (FTO^cko^), in which FTO expression was specifically abolished in meniscus cells by crossing FTO^flox/flox^ mice with COL1A1‐CreER^T2^ mice (**Figure** **4**A). The genotypes of FTO^cko^ were determined by PCR (Figure S5A, Supporting Information). DMM surgery was also performed in control and FTO^cko^ mice and tamoxifen was injected intraperitoneally five times 1 week after surgery (Figure 4B). The weights of the control and FTO^cko^ mice did not significantly differ after tamoxifen treatment (Figure S5B, Supporting Information). FTO was also validated to be significantly deleted in FTO^cko^ mice (Figure S5C, Supporting Information). No significant differences in gross appearance or body length were observed at the age of 4 months between control and FTO^cko^ mice (Figure S6A, Supporting Information). At the age of 12 months, the gross appearance and length of the FTO^cko^ mice resembled those of the control mice (Figure S6B, Supporting Information). Similarly, no significant differences were detected in the length of the hindlimbs between control and FTO^cko^ mice (Figure S6C,D, Supporting Information).

3D micro‐CT analysis revealed significantly more osteophytes in FTO^cko^ mice that underwent DMM surgery (Figure 4C,D). Much more severe cartilage destruction and proteoglycan loss were observed in meniscus from DMM‐induced OA mice with FTO deletion, which was consistent with the OARSI grading, and meniscus score evaluation also revealed significantly severe meniscus degeneration in the FTO^cko^ mice in the sham and DMM groups (Figure 4E). HE staining also revealed aggravated synovial inflammation in FTO^cko^ mice (Figure 4F). IF staining of m^6^A showed that the m^6^A ratio was greater in the meniscus and cartilage of FTO^cko^ mice that underwent DMM surgery than in that of control mice (Figure 4G). Consistently, the ratio of ACAN was decreased in the cartilage of FTO^cko^ mice that underwent DMM surgery, while the ratio of MMP13 was increased (Figure S5D, Supporting Information). The intensity of COL1A1 in the meniscus was markedly decreased in FTO^cko^ mice in the DMM group, and the number of MMP3‐positive cells was increased in FTO^cko^ mice (Figure 4H,I). Additionally, the ratio of LC3B‐positive cells was prominently lower in FTO^cko^ mice that underwent DMM surgery, and the ratio of p62‐positive cells showed the opposite trend (Figure 4H,I). Hence, specific knockout of FTO in meniscus cells in mice promoted meniscus degeneration and OA in DMM‐induced OA model.

The knee joints of 12‐month‐old FTO^cko^ mice and control mice were also collected. HE and SOFG staining were performed. The meniscus score was increased in 12‐month‐old FTO^cko^ mice, as was the OARSI score, indicating cartilage degeneration (Figure S6E,F, Supporting Information). IF staining revealed that the intensity of COL1A1 and LC3B was significantly decreased in aging FTO^cko^ mice, while the intensity of MMP3 and p62 was increased (Figure S6G,H, Supporting Information).

### 2.6 FTO Mediated the Expression of ATG16L1 in an m^6^A‐Dependent Manner

**Figure 5:**
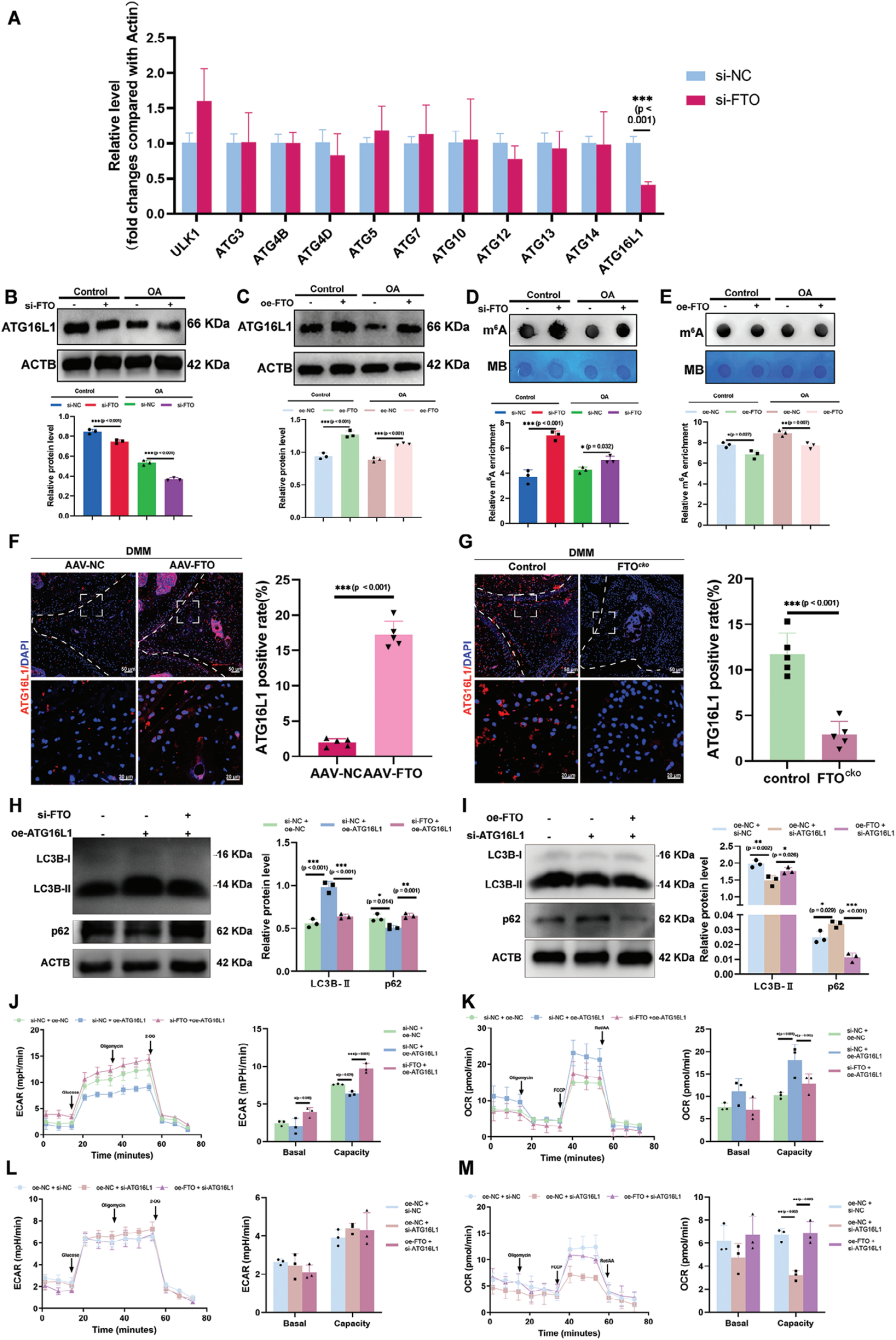
FTO modified autophagy and bioenergetic metabolism by regulating the expression of ATG16L1. A) qRT‒PCR analysis of several classic autophagy‐related proteins (ATGs) in meniscal cells with and without si‐FTO transfection. B,C) The protein levels and quantification of ATG16L1 in meniscus cells transfected with or without small interfering RNA (si‐FTO) and plasmids overexpressing FTO (oe‐FTO). *n* = 3 per group. D,E) Dot blot analysis of m^6^A in meniscus cells transfected with small interfering RNA knocking down FTO (si‐FTO) or with plasmids overexpressing FTO (oe‐FTO). *n* = 3 per group. F) Representative IF image of ATG16L1 in the anterior meniscus of mice subjected to DMM surgery treated with or without AAV‐FTO. *n* = 5 per group. G) Representative IF image of ATG16L1 and quantification of ATG16L1 in the anterior meniscus of control and FTO^cko^ mice that underwent DMM surgery. *n* = 5 per group. H) WB analysis and protein levels of LC3B and p62 in meniscus cells treated with si‐NC + oe‐NC, si‐NC + oe‐ATG16L1, and si‐FTO + oe‐ATG16L1. I) WB analysis and protein levels of LC3B and p62 in meniscus cells treated with si‐NC + oe‐NC, oe‐NC + si‐ATG16L1, and oe‐FTO + si‐ATG16L1. J,K) ECAR and OCR analysis of meniscus cells treated with si‐NC + oe‐NC, si‐NC + oe‐ATG16L1, and si‐FTO + oe‐ATG16L1. *n* = 3 per group. L,M) ECAR and OCR analysis of meniscus cells treated with si‐NC + oe‐NC, oe‐NC + si‐ATG16L1, and oe‐FTO + si‐ATG16L1. *n* = 3 per group. All quantification data are presented as the mean ± SEM. F,G) Unpaired Student's *t*‐test and B–E,H–M) one‐way ANOVA followed with Turky’ HSD test were used for statistical analysis and *p*‐values are presented in each bar plot.

After validating the function of FTO in vitro and in vivo, we further investigated the underlying mechanism of the m^6^A methyltransferase FTO in autophagy and energy metabolism. We evaluated the expression levels of several classic autophagic markers after knocking down FTO in meniscus cells.^[^ ^36^ ^]^ Among the other autophagy‐related genes, ATG16L1, one of the main components of the ATG2‐ATG5‐ATG16L1 complex,^[^ ^37^ ^]^ was the most significantly downregulated after transfection with si‐FTO (**Figure** **5**A). The ratio of ATG16L1 was significantly decreased in the meniscus tissues and meniscus cells from OA patients (Figure S7A,B, Supporting Information). The expression of ATG16L1 in the meniscus was also downregulated in aging mice and DMM‐induced OA mice (Figure S7C,D, Supporting Information). Consistently, transfection with si‐FTO decreased the protein level of ATG16L1 (Figure 5B). On the other hand, overexpression of FTO enhanced the expression of ATG16L1 in meniscus cells. (Figure 5C). In terms of m^6^A modification, m^6^A dot blot assays showed that m^6^A methylation was significantly enhanced after knocking down FTO, while overexpressing FTO decreased the intensity of m^6^A methylation (Figure 5D,E). More importantly, overexpression of FTO using AAV promoted the expression of ATG16L1 in DMM mice (Figure 5F). Conditional knockout of FTO in meniscus cells significantly downregulated the expression of ATG16L1 in FTO^cko^ mice in the DMM model (Figure 5G). Silencing ATG16L1 in meniscus cells can significantly decreased the expression of COL1A1, while increased the levels of MMP3 (Figure S7E, Supporting Information). Cotransfection of oe‐ATG16L1 and si‐FTO in meniscus cells significantly downregulated the expression of LC3B‐II and upregulated the expression of p62 compared with solely transfection of oe‐ATG16L1 (Figure 5H). On the other hand, the levels of LC3B‐II were increased and the levels of p62 were decreased in meniscus cells cotransfected with si‐ATG16L1 and oe‐FTO compared with cells transfected with si‐ATG16L1 alone (Figure 5I). Moreover, overexpression of ATG16L1 alleviated lactate production and glycolysis, while silencing FTO and overexpression of ATG16L1 simultaneously enhanced the ECAR in meniscus cells. (Figure 5J). The rate of mitochondrial respiration was also decreased in meniscus cells transfected with si‐FTO and oe‐ATG16L1 compared with cells transfected with oe‐ATG16L1 alone (Figure 5K). In contrast, cotransfection of oe‐FTO and si‐ATG16L1 resulted in increased glycolytic rates and decreased mitochondrial respiration compared with transfected with si‐ATG16L1 alone (Figure 5L,M). These findings suggested that FTO mediated autophagy‐related energy metabolism by regulating ATG16L1 in an m^6^A‐dependent manner.

### 2.7 FTO Mediated the m^6^A Modification of ATG16L1 in a YTHDF2‐Dependent Manner

**Figure 6:**
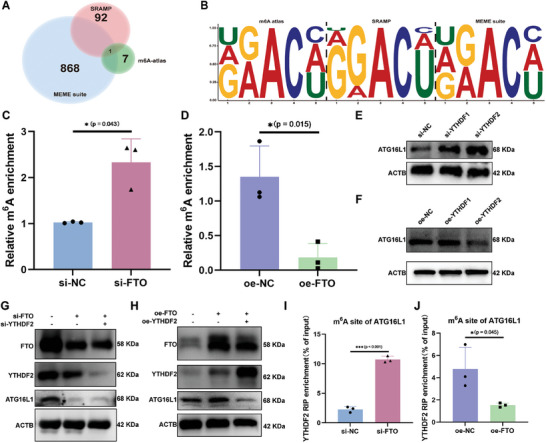
FTO regulates ATG16L1 through m^6^A in a YTHDF2‐dependent manner. A) Venn diagram of potential m^6^A sites on ATG16L1 mRNA transcripts according to the overlapping MEME suite, m^6^A atlas, and SRAMP. B) The potential sites in ATG16L1 mRNA transcripts predicted via the MEME suite, the m^6^A atlas, and the SRAMP database. C) MeRIP‐qPCR analysis of m^6^A enrichment on the ATG16L1 mRNA transcript in meniscus cells transfected with and without si‐FTO. *n* = 3 per group. D) MeRIP‐qPCR analysis of m^6^A enrichment on ATG16L1 mRNA transcripts in meniscus cells transfected with or without oe‐FTO. *n* = 3 per group. E) The WB analysis of ATG16L1 in meniscus cells treated with si‐YTHDF1 and si‐YTHDF2. *n* = 3 per group. F) The WB analysis of ATG16L1 in meniscus cells treated with oe‐YTHDF1 and oe‐YTHDF2. *n* = 3 per group. G) The WB analysis of FTO, YTHDF2, and ATG16L1 in meniscus cells treated with si‐NC, si‐ FTO, and si‐FTO + si‐YTHDF2. H) WB analysis of FTO, YTHDF2, and ATG16L1 in meniscus cells in treated with oe‐NC, oe‐FTO, and both oe‐FTO and oe‐YTHDF2. I) RIP‐qPCR analysis of enrichment of YTHDF2 on the predicted m^6^A site of ATG16L1 in meniscus cells treated with and without si‐FTO transfection. *n* = 3 per group. J) RIP‐qPCR analysis of the enrichment of YTHDF2 on the predicted m^6^A site of ATG16L1 in meniscus cells treated with and without oe‐FTO. *n* = 3 per group. All quantification data are presented as the mean ± SEM. Unpaired Student's *t*‐test (Figure 5C,D,I,J) was performed and *p*‐values are presented in each bar plot.

To determine how FTO modulates the expression of ATG16L1 via m^6^A modification, we first screened out the potential site of m^6^A methylation on the transcript of ATG16L1. Classic online motif‐based sequence analysis tool like MEME suite,^[^ ^38^ ^]^ SRAMP,^[^ ^39^ ^]^ and m^6^A atlas^[^ ^40^ ^]^ were used to predict the site of m^6^A methylation on ATG16L1, and surprisingly, only 1 site, GAACU, was identified as a potential site of m^6^A methylation (**Figure** **6**A,B). To validate whether FTO regulates the m^6^A methylation of the messenger RNA of ATG16L1, methylated RNA immunoprecipitation quantitative PCR (meRIP‐qPCR) assays were performed, and the results showed that silencing FTO by transfection with si‐FTO increased the m^6^A levels in the mRNA transcript of ATG16L1 (Figure 6C). On the other hand, FTO overexpression significantly decreased the m^6^A level in ATG16L1 (Figure 6D). m^6^A methylation is also regulated by specific m^6^A reader proteins, such as YTH N6‐methyladenosine RNA binding protein 1/2 (YTHDF1/2).^[^ ^41^ ^]^ To study whether the downregulation of FTO in meniscus degeneration modulates m^6^A methylation at the ATG16L1 transcript via YTHDF1 or YTHDF2, we first tested the protein levels of ATG16L1 after transfection with si‐YTHDF1, si‐YTHDF2, oe‐YTHDF1, or oe‐YTHDF2 alone. The protein levels of ATG16L1 were upregulated after YTHDF2 knockdown, while silencing YTHDF1 had no significant effect on the ATG16L1 levels (Figure 6E). Overexpressing YTHDF2 decreased the protein level of ATG16L1, while YTHDF1 overexpression had no significant effect (Figure 6F). Moreover, we performed cotransfection to validate whether YTHDF2 was necessary for m^6^A methylation under FTO regulation. Compared with cotransfection with si‐FTO alone, cotransfection with si‐FTO and si‐YTHDF2 increased the level of ATG16L1 (Figure 6G). In contrast, cotransfection of oe‐FTO and oe‐YTHDF2 significantly downregulated ATG16L1 levels (Figure 6H). RNA‐immunoprecipitation followed by qPCR revealed that silencing FTO increased the enrichment of YTHDF2 on ATG16L1, while overexpressing FTO decreased it (Figure 6I,J).

### 2.8 FTO Overexpression Alleviates Meniscus Degeneration and Decreases OA Severity in FTO^cko^ Mice

**Figure 7:**
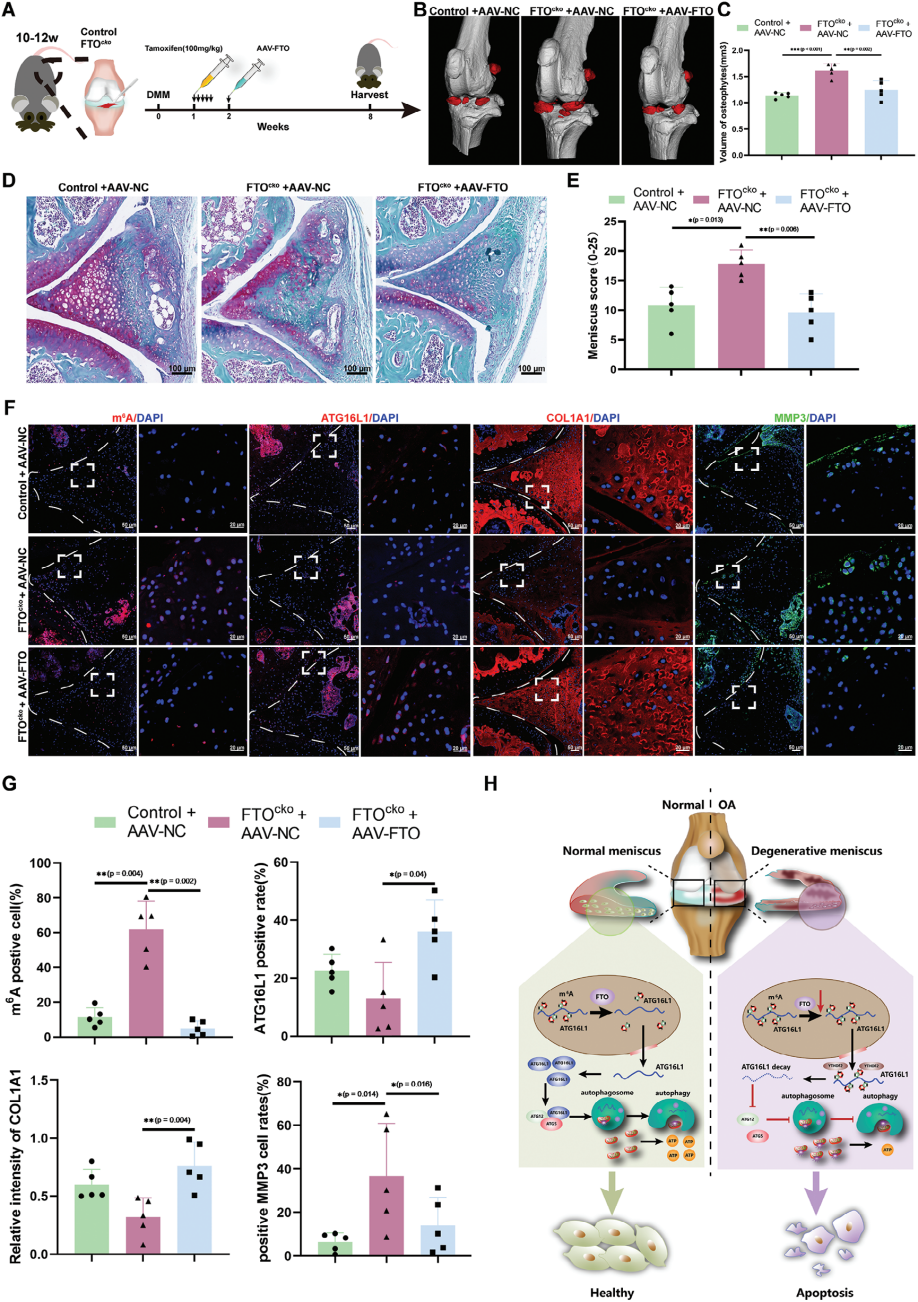
Intra‐articular injection of AAV‐FTO alleviates meniscus degeneration in FTO^cko^ mice. A) Schematic illustration of DMM surgery and intra‐articular injection of AAV‐FTO in FTO^cko^ mice, which were grouped as the control + AAV‐NC, FTO^cko^ + AAV‐NC, and FTO^cko^ + AAV‐FTO groups. *n* = 5 per group. B) Representative micro‐CT reconstruction and identification of osteophytes (red) in the control + AAV‐NC, FTO^cko^ + AAV‐NC, and FTO^cko^ + AAV‐FTO groups. C) Quantification and analysis of the volume of osteophytes. *n* = 5 per group. D,E) Representative SOFG of the cartilage and meniscus in the Control + AAV‐NC, FTO^cko^ + AAV‐NC, and FTO^cko^ + AAV‐FTO groups, along with the evaluation of meniscus injury based on the meniscus score. *n* = 5 per group. F) Representative IF images of COL1A1 (red), MMP3 (green), m^6^A (red), and ATG16L1 (red) in the anterior meniscus of control + AAV‐NC, FTO^cko^ + AAV‐NC, and FTO^cko^ + AAV‐FTO mice. *n* = 5 per group. G) Quantification of the intensity of COL1A1 and the percentages of MMP3‐, m^6^A‐, and ATG16L1‐positive cells in the anterior meniscus of control + AAV‐NC, FTO^cko^ + AAV‐NC, and FTO^cko^ + AAV‐FTO mice. *n* = 5 per group. H) Schematic representations of the mechanisms by which FTO/m^6^A/ATG16L1 mediates autophagy and bioenergetic metabolism in meniscus cells. All quantification data are presented as the mean ± SEM. C,E,G) One‐way ANOVA followed with Turky’ HSD test was used for statistical analysis and *p*‐values are presented in each bar plot.

In vivo rescue studies were conducted in FTO^cko^ mice with DMM‐induced OA by intra‐articular injection of AAV‐FTO 4 times consecutively per week after specific deletion of FTO by tamoxifen induction five times 1 week postsurgery (**Figure** **7**A). Micro‐CT reconstruction of the knee joints revealed that the volume of osteophytes was significantly lower in the FTO^cko^ mice injected with AAV‐FTO (FTO^cko^ + AAV‐FTO) than in the control group (FTO^cko^ + AAV‐NC (normal control)) (Figure 7B,C). SOFG staining revealed that meniscus damage and cartilage degeneration were significantly alleviated in the FTO^cko^ + AAV‐FTO group compared with the FTO^cko^ + AAV‐NC group (Figure 7D,E and Figure S8A,B, Supporting Information). Compared with those in the FTO^cko^ + AAV‐NC group, the percentages of m^6^A‐ and MMP3‐positive meniscus cells in the knee joints were markedly lower in the FTO^cko^ + AAV‐FTO group, while the intensity of COL1A1 and the percentage of ATG16L1‐positive cells were greater in the FTO^cko^ + AAV‐FTO group (Figure 7F,G).

## 3 Discussion

Abnormal bioenergetic metabolism was shown to be regulated by impaired autophagy during meniscus degeneration in OA. FTO, a methyltransferase of m^6^A, is downregulated in meniscus degeneration, and the function of FTO in regulating dysregulated autophagy and bioenergetic metabolism has been studied in vitro and in vivo. Specifically, FTO regulated ATG16L1 in an m^6^A‐dependent manner. In this study, we discovered that dysregulation of FTO/m^6^A/ATG16L1 is a key regulator of impaired autophagy and bioenergetic metabolism during meniscus degeneration in OA.

Dysfunctional energy metabolism has been implicated in diseases related to aging, such as Alzheimer's disease.^[^ ^42^ ^]^ Enhanced glycolysis was detected in chondrocytes with OA.^[^ ^15^ ^]^ Restoring the balance between glycolysis and the tricarboxylic acid cycle could protect the integrity of cartilage and alleviate OA.^[^ ^43^ ^]^ A photosynthetic system also showed promises in curing OA by recovering the energy metabolism in chondrocytes.^[^ ^44^ ^]^ In this study, we aimed to determine the bioenergetic metabolic patterns in meniscus cells with and without OA. Targeted metabolomic analysis suggested that meniscal cells with OA were enriched in glycolysis metabolites and pathway enrichment analysis also revealed enhanced aerobic glycolysis during OA in meniscus cells. However, enhanced aerobic glycolysis was also observed in normal chondrocytes under oxygen tension, suggesting that aerobic glycolysis might act as a compensatory bioenergetic metabolic pathway under biological stress. Based on previous studies, dysfunctional energetic metabolic organelles such as mitochondria might be the main cause of dysregulated bioenergetic metabolism in OA.^[^ ^10^, ^45^ ^]^ Moreover, mitochondrial OXPHO, along with abnormal mitochondrial microstructure and function, was also inhibited in meniscus cells from patients with OA. These findings suggested that the metabolic shift from OXPHO to glycolysis might be due to certain dysfunctional bioenergetic organelles.

The regulation of autophagy is also related to the progression of OA.^[^ ^29^, ^46^, ^47^ ^]^ Autophagy is an important biological process for engulfing dysfunctional organelles and enzymes and providing substrates for metabolism.^[^ ^48^ ^]^ The main function of autophagy is to restore the homeostasis of individual cells by performing quality control via selectively degrading impaired organelles.^[^ ^49^ ^]^ Similarly, autophagy plays an important role in modulating the phagocytosis of biomacromolecules such as senescent cell to prevent production of proinflammatory and matrix‐degrading molecules, also known as senescence‐associated secretory phenotype.^[^ ^22^ ^]^ Intriguingly, autophagic process might also activates or aggravates OA via diverse biological process. For instance, autophagosomes engulfing minerals initiates calcification of collagen hydrogel in cartilage, which suggests a potential mechanism of the onset of early‐stage OA.^[^ ^50^ ^]^ Enhancing release of cathepsin B caused by phagocytosis of hydroxyapatite crystallites in cartilage results in activation of NLR family pyrin domain containing 3 inflammasome‐related pyroptosis, thereby induces cartilage degeneration, showing a vicious circle of cartilage calcification and cartilage degeneration linked by autophagy and phagocytosis.^[^ ^51^ ^]^ Hence, it is still debatable that to what extent that autophagy might serves as a protective role for OA. Our findings revealed that autophagy is inhibited in meniscus cells from patients with OA and mice with age‐related or DMM‐induced OA model, which is consistent with the findings of previous studies.^[^ ^20^ ^]^ Next, we sought to determine whether impaired autophagy activates metabolic shifts during meniscal degeneration. Autophagy inhibition by 3‐MA significantly suppressed the oxygen consumption rate (OCR) in meniscus cells, while rapamycin treatment restored the ratio of OXPHO and ATP production in meniscus cells. However, both 3‐MA and RAPA stimulation showed enhanced ECAR capacity. Previous studies suggested that Inhibition of autophagy using 3‐MA may modulates the levels of enzymes in metabolic pathways or offered essential components for metabolism, leading to a temporary reliance on glycolysis to meet the energy demands of cells.^[^ ^52^, ^53^, ^54^ ^]^ This can cause sustainment and enhancement of ECAR to a similar extent as autophagy induction. Similarly, in some cases, autophagy activation with RAPA may enhance organelles clearance and promote metabolic reprogramming toward glycolysis, which may also result in increased ECAR.^[^ ^55^ ^]^ The enhanced ECAR capacity upon 3‐MA and RAPA treatments might reflect the interplay between autophagy and glycolysis in maintaining cellular energy homeostasis. Upon autophagic inhibition and activation, meniscus cells potentially rely on glycolysis to conduct metabolic adaptation, leading to comparable metabolic outcomes. Overall, these results suggest that autophagy regulates bioenergetic metabolism in meniscus cells and might act as an important biological process during meniscal degeneration. Interestingly, recent studies also suggest that meniscal calcification serves as an important pathological phenomenon in meniscus degeneration.^[^ ^56^ ^]^ Whether autophagy relates with meniscal calcification during meniscus degeneration and worths further investigation.

m^6^A modification has been suggested to be involved in multiple biological processes by regulating the stability of messenger RNA, including energetic metabolism and autophagy. This widespread epitranscriptomic regulatory mechanism has been reported to regulate inflammation, cellular senescence and cell differentiation.^[^ ^57^, ^58^, ^59^ ^]^ Previous studies have shown that m^6^A modification might be an important mechanism in the homeostasis of chondrocytes,^[^ ^60^ ^]^ mesenchymal stem cells (MSCs),^[^ ^61^ ^]^ and FLSs.^[^ ^22^ ^]^ In our study, we detected elevated m^6^A levels in meniscus cells from patients with OA and the expression of FTO was the most differentially downregulated among the m^6^A “writers” and “erasers”. Previous studies have shown that FTO might regulate metabolic reprogramming in an m^6^A‐dependent manner to maintain the mRNA stability of specific glycolytic enzymes.^[^ ^62^ ^]^ In the aspect of regulating autophagy, the expression level of FTO could serve as the link between the m^6^A methylation and autophagy. He et al. demonstrated that impaired autophagic lysosomal process caused by arsenic exposure resulted in stabilized FTO expression, leading to another feedback to regulate autophagy.^[^ ^63^ ^]^ Consistently, our research demonstrated that the downregulation of FTO in meniscus cells with OA diminished autophagy and induced an aberrant metabolic shift, which resulted in decreased ATP production. In a mouse model of DMM‐induced OA, intra‐articular injection of AAV‐FTO reversed the inhibition of autophagy in DMM‐induced OA mice and alleviated meniscus degeneration. Specific knockdown of FTO in meniscus cells in vivo by generating FTO^cko^ mice. In the FTO^cko^ mouse model of OA, meniscus degeneration and OA progression were significantly accelerated by inhibiting autophagy in meniscus cells. Hence, we propose that the downregulation of FTO impairs autophagy and energetic metabolism in meniscus cells, which suggests that FTO is a novel target for the treatment of meniscal degeneration.

M^6^A demethyltransferase FTO is essentially necessary in regulating energetic metabolism and autophagy flux, but the specific mechanism is extremely complex. According to previous studies, FTO sustains the mRNA stability of autophagy‐related genes 5 (ATG5) and ATG7 via YTHDF2 in a m^6^A‐dependent manner, plays a critical role in the formation of autophagosome and thereby maintaining the interplay between autophagy and adipogenesis.^[^ ^24^ ^]^ YTHDF2 has also been known as an m^6^A‐specific binding “reader” for m^6^A methylation.^[^ ^64^, ^65^ ^]^ On the other hand, FTO could also stabilized the mRNA stability of p62, a classic autophagic substrate, to regulate autophagy.^[^ ^27^ ^]^ However, whether FTO modulate LC3, the marker for autophagosome is still unclear. Though FTO has also been studied as an important epigenetic regulator of OA, the specific mechanism of FTO regulating the axis of autophagy‐energetic metabolism remains enigmatic. Our study discovered that ATG16L1 was the most differentially expressed gene under si‐FTO transfection. ATG16L1 is one of the most necessary components of the ATG12‐ATG5‐ATG16L complex and participates in vesicle elongation during the expansion and completion of autophagosomes.^[^ ^17^, ^66^ ^]^ It has been widely considered as an important regulator in Crohn's Diseases because of T300A polymorphism.^[^ ^67^ ^]^ The dynamics of ATG16L1 expression level regulates the formation of autophagosomes, hence relates with inflammation^[^ ^68^ ^]^ and immune response.^[^ ^69^, ^70^ ^]^ In our present study, the decreased levels of ATG16L1 caused by the downregulation of FTO might be a possible cause of impaired autophagosome formation and aberrant energy metabolism in meniscus degeneration. We validated that the downregulation of FTO resulted in lower levels of ATG16L1 in meniscus cells from patients with OA and enhanced m^6^A methylation. Through rescue experiment by cotransfection, FTO was proved to rescue the levels of ATG16L1 and restore autophagy and energetic metabolism. Additionally, WB showed that YTHDF2 significantly regulated the levels of ATG16L1 when cotransfected with si‐FTO or oe‐FTO, while YTHDF1 had no effect on ATG16L1. Moreover, the overexpression of FTO in FTO^cko^ mice subjected to DMM surgery showed alleviated meniscus destruction and cartilage degeneration, with decreased m^6^A methylation and restored ATG16L1 levels in meniscus cells. Therefore, FTO/m^6^A/ATG16L1 axis regulates the formation of autophagosomes, specifically in the ATG16L1‐regulated vesicle elongation, thereby maintaining the homeostasis of autophagy and energetic metabolism in meniscus.

There were also several limitations in this study. First, a comprehensive database of degenerated meniscal tissue from patients with different degrees of OA is difficult to construct due to difficulties in sample collection. Additionally, for technical reasons, we have not screened potential therapeutic drugs for maintaining the regulation of the FTO/m^6^A/ATG16L1 axis, which we plan to perform in our future studies.

To summarize, the findings of the present study demonstrated that dysregulation of the FTO/m^6^A/ATG16L1 axis impairs autophagy in meniscus cells, thereby promoting bioenergetic metabolic disorders and accelerating meniscus degeneration and OA (Figure 7h). Our study highlights the importance of m^6^A methylation in regulating autophagy and bioenergetic metabolism in meniscus degeneration and OA and provides insight into the mechanism of meniscus degeneration and novel therapeutic strategies for meniscus degeneration and OA.

## 4 Experimental Section

### Human Samples

Human OA menisci were harvested from patients who were diagnosed with OA and underwent total knee arthroplasty (*n* = 10, aged 55–70 years). Normal meniscus tissue from the control group was collected from patients who underwent amputation due to deadly trauma and who had no history of arthritis (*n* = 10, aged 25–35 years). Informed consent from both patients and the approval of the Ethics and Committee of the First Affiliated Hospital of Sun Yat‐sen University (Guangzhou, China) were obtained before the human meniscus specimens were harvested. All procedures were performed with Ethical Committee of the First Affiliated Hospital of Sun Yat‐sen University ([2021]667). Each tissue sample was subjected to hematoxylin‐eosin, Safranin O/Fast green, IF, and immunohistochemistry (IHC) staining.

### Targeted‐Metabolomics of Energetic Metabolism

Primary human meniscus cell samples were isolated and frozen in liquid nitrogen using a mortar and pestle. Each sample was homogenized in 1 mL of cold methanol/acetonitrile/H2O solution (2:2:1, v/v/v) and sonicated twice for 30 min (twice) at low temperature. The homogenates were centrifuged at 14 000 g for 20 min at 4 °C The resulting supernatants were vacuum dried in a centrifuge. For liquid chromatograph‐mass spectrometer (LC‐MS) analysis, each dried sample was redissolved in 100 mL of acetonitrile/H2O solution (1:1, v/v), vortexed and then centrifuged at 14 000 rpm for 15 min at 4 C. The supernatant was analyzed using LC‐MS/MS. Targeted metabolic analysis was performed using an LC‐MS/MS system (Shanghai Applied Protein Technology Co., Ltd.).

### Interleukin‐1β(IL‐1β)‐Induced OA Model

The construction of IL‐1β‐induced OA model in meniscus cells was carried out as previously described. In brief, 10 ng mL^−1^ IL‐1β was dissolved with Dulbecco's modified Eagle's medium (DMEM)/Nutrient

Mixture F‐12 (Gibco Life Technologies, Grand Island, NY, United States), 5% fetal bovine serum (FBS; Gibco Life Technologies), and 100 IU mL^−1^ of penicillin (PS; Gibco Life Technologies) and incubated with primary meniscus cells for 48 h at 37 °C in a humidified atmosphere of 5% CO_2_ and 1% oxygen.

### Mice

FTO^flox/flox^ mice were purchased from Shanghai Model Organisms, and COL1A1‐CreER^T2^ mice were purchased from Jackson Laboratory (Bar Harbor, ME, USA). COL1A1‐CreER^T2^ mice and FTO^flox/flox^ mice were mated to produce FTO^flox/+^; COL1A1‐CreER^T2^ mice, which were then mated with FTO^flox/flox^ mice to recreate homozygous FTO^flox/flox^; COL1A1‐CreER^T2^ mice. The identification of genotypes was performed according to the manufacturer's instructions. All mice were provided food and water under pathogen‐free conditions at constant temperature (23–25 °C) and stable humidity (50–60%), with five or fewer mice per cage. Both animal experiments were approved by the Committee of Animal Care and Use Committee of Sun Yat‐sen University (SYSU‐IACUC‐2023‐000399).

### Animal Model

Ten‐ to 12‐week‐old male C57BL/6J mice were purchased from Gempharmatech. All FTO^flox/flox^; COL1A1‐CreER^T2^ and FTO^flox/flox^ mice were injected intraperitoneally with tamoxifen (100 µg g^−1^) for five consecutive days following the protocols. One‐ to two‐year‐old C57BL/6J mice were selected as the aging mouse model. Both C57BL/6J mice and transgenic mice underwent DMM surgery to establish an experimental OA model. The protocol for DMM surgery was published in the previous studies.

### Intra‐Articular Injection of an Adeno‐Associated Virus Overexpressing FTO in an Experimental Mouse Model of OA

For intra‐articular delivery, 10 µL of rAAV9 or rAAV9‐FTO was intra‐articularly injected into the knee joints of mice once a week for four consecutive weeks. Eight weeks after DMM surgery, the knee joint was collected for subsequent testing.

### Isolation of Primary Meniscus Cells and Cell Culture

Normal healthy meniscus and OA degenerative meniscus tissues were both cut into slices and digested with 2 mg mL^−1^ collagenase P (Roche) overnight. Specifically, meniscus cells in the vascularized area were cut from the outer 1/3 region of the meniscus and cells in the avascularized area were sliced from the inner region of the meniscus tissue. The isolated cells were then cultured in DMEM/F‐12 supplemented with 10% FBS. The medium was then changed after 1 d of culture to remove nonadherent cells. The cells were then incubated at 37 °C with 5% CO_2_ until they reached 90% confluence.

### Construction and Transfection of siRNAs and Plasmids

Small interfering RNA synthesis of FTO and ATG16L1 was performed by RiboBio. The sequences of each siRNA used were as follows: the FTO and ATG16L1 genes were cloned and inserted into the pcDNA 3.1 vector at HanBio. Both siRNAs and plasmids were transfected into primary meniscus cells by using Lipofectamine 3000 (Invitrogen) according to the manufacturer's protocols.

### Seahorse Real‐Time Cell Energy Metabolism Analysis

The dynamic energy metabolism phenotype of the meniscus cells was determined by mitochondrial stress tests and glycolysis stress tests using a Seahorse XF Cell Mito Stress Kit (103010‐100) and a Seahorse XF Cell Glycolysis Stress Kit (103020‐100), respectively. A total of 20 000 meniscus cells were plated in 96‐well Seahorse Cell Culture Plates per well. Before energy metabolism analysis, the Seahorse XFe96 Sensor Cartridges were preheated and hydrated with DNase/RNase‐free water overnight at 37 °C without CO_2_ supplementation, and XF Calibration Solution was added to each well. For the mitochondrial stress test, oligomycin, carbonyl cyanide 4‐(trifluoromethoxy)phenylhydrazone (FCCP), and antimycin A/rotenone were added sequentially to each well and the OCR was determined with a Seahorse XFe96 analyzer. Similarly, glucose, 2‐deoxyglucose, and oligomycin were successively added to each well to induce glycolytic stress and the ECAR was quantified with an XFe96 analyzer.

### TEM

Primary meniscus cells were obtained, fixed with 2.5% glutaraldehyde, rinsed with phosphate buffered saline (PBS), postfixed with 1% osmium tetroxide, and dehydrated using ethanol. Subsequently, the samples were embedded in araldite and ultrathin slices were prepared and double‐stained with uranyl acetate and lead citrate using standard techniques. Finally, a transmission electron microscope (FEI Tecnai G2 Spirit Twin) was used to capture high‐resolution images.

### Measurement of Mitochondrial Membrane Potential (MMP), MitoSOX, and Intracellular ROS Levels

The MMP was detected using an enhanced MMP assay kit with JC‐1 (Beyotime). Mitochondrial superoxide levels in living cells were measured using the MitoSOX mitochondrial superoxide indicator (Thermo Fisher Scientific). Total intracellular levels of ROS were quantified using an ROS assay kit (Beyotime). A flow cytometer (Beckman Coulter) or a laser scanning confocal microscope (LSM780) was used for observation.

### Total RNA Extraction and qRT‒PCR Analysis

Total RNA was extracted from human meniscus tissue and primary human meniscus cells using TRIzol reagent (Invitrogen) and a total RNA extraction kit (Qiagen). After reverse transcription into cDNA, qPCR was performed using SYBR Premix Ex Taq II (TaKaRa Bio, Japan). The primers used for the analysis are listed in Table S3 in the Supporting Information. The data were analyzed following the 2−ΔΔ*t* method, with β‐actin used as the reference gene.

### Western Blot Analysis

Meniscus cells were lysed on ice using radio immunoprecipitation assay (RIPA) lysis buffer (Beyotime) containing a phenylmethylsulfonyl fluoride (PMSF) protease inhibitor cocktail to collect the supernatants and then a bicinchoninic acid (BCA) Protein Assay Kit (Thermo Fisher) was used to measure the concentration. Total protein was further separated by sodium dodecyl sulfate‐polyacrylamide gel electrophoresis (SDS–PAGE) before being transferred to polyvinylidene difluoride (PVDF) membranes (Millipore). Then, the membranes were blocked and incubated with primary antibodies against COL1A1, MMP1, LC3B, p62, FTO, ATG16L1, and β‐actin overnight at 4 °C. Subsequently, the membranes were incubated with the corresponding horseradish peroxidase (HRP)‐conjugated secondary antibodies at room temperature for 1 h. Protein bands were visualized using enhanced chemiluminescence (ECL) solution (Millipore) and detected using an automatic chemiluminescence imaging analysis system (Bio‐Rad). ImageJ software (version v.1.51a) was used for subsequent semiquantitative analysis. For the quantification of LC3B‐II/LC3B‐I ratio, the intensities of LC3B‐II and LC3B‐I bands were normalized to a loading control (e.g., β‐actin) to account for variations in loading or transfer efficiency and lastly, the normalized intensity of LC3B‐II was divided by the normalized intensity of LC3B‐I.

### Histology

Tissue samples were fixed in 4% paraformaldehyde (PFA) overnight and embedded in paraffin after decalcification with ethylenediaminetetraacetic acid. Then, the wax blocks were cut into 6 µm thick sagittal slices. Fast Green/Safranin O and hematoxylin and eosin (H&E) staining were performed. Joint pathology was quantified using OARSI meniscus scoring.

### Tissue IF and IHC

For tissue IF, 6 µm thick paraffin mouse meniscus sections were immersed in xylene and graded ethanol to deparaffinize and regain water before antigen retrieval in citric acid buffer (pH 6.0). Next, the samples were further incubated with 3% H_2_O_2_ and blocked with goat serum. Subsequently, the sections were incubated with primary antibodies overnight at 4 °C and incubated with the corresponding secondary antibodies for 1 h at room temperature, followed by incubation with 4',6‐diamidino‐2‐phenylindole (DAPI). Images were visualized under a confocal laser microscope (LSM780).

For tissue IHC, human meniscal tissue sections were dewaxed, hydrated, subjected to antigen retrieval, blocked as described above, incubated with the corresponding primary antibodies overnight at 4 °C and then incubated with the appropriate secondary antibodies for 1 h. Antigen‐positive cells were visualized using a DAB substrate kit (ZSGB‐BIO).

### Methylated RNA Immunoprecipitation‐PCR (m^6^A RIP‐qPCR)

A magna MeRIP m^6^A kit (Millipore Sigma) was used to measure the m^6^A modification levels in the mRNA of ATG16L1. According to the manufacturer's instructions, the isolated RNA was fragmented, 1/10 of the total RNA was saved as the input RNA, and the remaining RNA was immunoprecipitated with magnetic beads A/G coated with m^6^A antibody overnight at 4 °C. After three washes, the m^6^A‐modified RNAs were eluted and recovered by ethanol precipitation to determine the m^6^A enrichment by qPCR. Moreover, the EpiQuik m^6^A RNA Methylation Quantification Kit (Epigentek) was used to detect the total cellular m^6^A levels.

### m^6^A Dot Blot Assay

Total RNA or poly(A) + mRNA was isolated as described above. mRNA samples dissolved in three volumes of RNA incubation buffer were denatured at 65 °C within 5 min. Then, the samples, which were divided into subgroups of 400, 200, and 100 ng, were loaded onto an Amersham Hybond‐N+ membrane (GE Healthcare, USA) installed in a BioDot Apparatus (Bio‐Rad, USA) with a mixture of ice‐cold 20× saline‐sodium citrate (SSC) buffer (Sigma‐Aldrich, Germany). The membrane was UV crosslinked for 5 min and washed with PBST. Then, the samples were stained with 0.02% methylene blue (Sangon Biotech, China), followed by scanning to indicate the total content of the input RNA. After being blocked with 5% nonfat milk, the membrane was incubated with a specific m^6^A antibody (1:1000, Millipore) overnight at 4 °C. Dot blots were incubated with HRP‐conjugated antimouse immunoglobulin G (IgG) for 1 h before visualization by an imaging system (Bio‐Rad, USA).

### RNA‐Immunoprecipitation (RIP)‐qPCR Analysis

RIP analysis was performed by using EZ‐Magna RIP RNA‐Binding Protein Immunoprecipitation Kit (Millipore Sigma) according to the instruction of the manufacturer. After isolating the input and immunoprecipitated RNAs, both were Purified by TRIzol reagent, and the fold enrichment was detected by qPCR.

### Statistical Analysis

All experiments were performed in triplicate. The results were analyzed using SPSS software (version 26.0) and are expressed as the mean ± standard deviation. Statistical significance was calculated using unpaired, two‐tailed student's *t*‐test for comparisons between two groups and one‐way analysis of variance (ANOVA) followed by Turky's honestly significant difference (HSD) test for multiple group comparisons. *P*‐values were considered significant at *p < 0.05, **p < 0.01, ***p < 0.001.

## Conflict of Interest

The authors declare no conflict of interest.

## Supporting information



Supporting Information

## Data Availability

Research data are not shared.
