# Supplementary material for: M6A Demethyltransferase FTO Attenuates Meniscus Degeneration and Osteoarthritis via Orchestrating Autophagy and Energetic Metabolism
Source: Adv Sci (Weinh). 2025 Jan 13;12(9):2412379. doi: 10.1002/advs.202412379 (PMC11884582; doi:10.1002/advs.202412379)
Supplement: Supplementary file 1 — Supporting Information [file ADVS-12-2412379-s001.docx]

**Supplemental information**

**M^6^A Demethyltransferase FTO Attenuates Meniscus Degeneration and Osteoarthritis via Orchestrating Autophagy and Energetic Metabolism**

Zongrui Jiang^1,2,3#^, Chengyun Zhang^1,3#^, Ruonan Liu^1,3#^, Zijing Zhu^1,3^, Dianbo Long^2,3^, Xingzhao Wen^1,3^, Zhijian Yang^1,3^, Dong Jiang^1,3^, Guping Mao^2,3^, Weiming Liao^1,3^, Zhiqi Zhang^1,3*^

**Figure S1.**


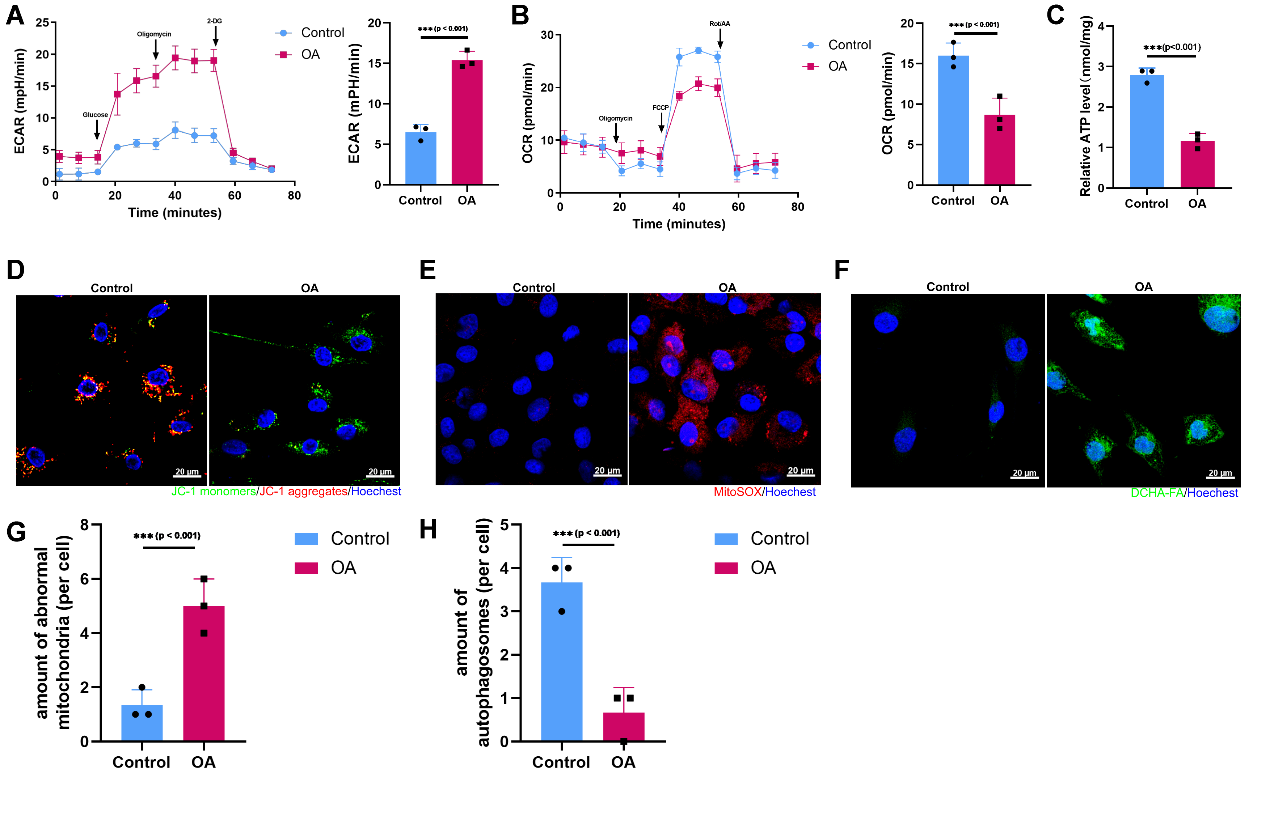


**Figure S1. Dysfunctional bioenergetic metabolism in meniscus cells with OA.**

(A and B) ECAR and OCR of meniscus cells from patients with and without OA were quantified by Seahorse XFe96 Real-time Analyzer n = 3.

(C) The quantification of ATP production in meniscus cells from patients with and without OA n = 3.

(D) Representative confocal images of JC-1 aggregates (red) and JC-1 monomers (green) in meniscus cells from patients with and without OA.

(E) The representative confocal images of MitoSOX (red) in meniscus cells with and without OA.

(F) The classic confocal images of DCFH-DA staining in meniscus cells with and without OA.

(G) The quantification of mitochondria with abnormal morphology in primary human meniscus cells, including swollen mitochondria n = 3.

(H) The quantification of autophagosome in primary human meniscus cells. n = 3.

All quantification data were presented the mean ± SEM. Unpaired t test was used for statistical analysis (A-C), and p values were presented in each bar plots.

**Figure S2.**


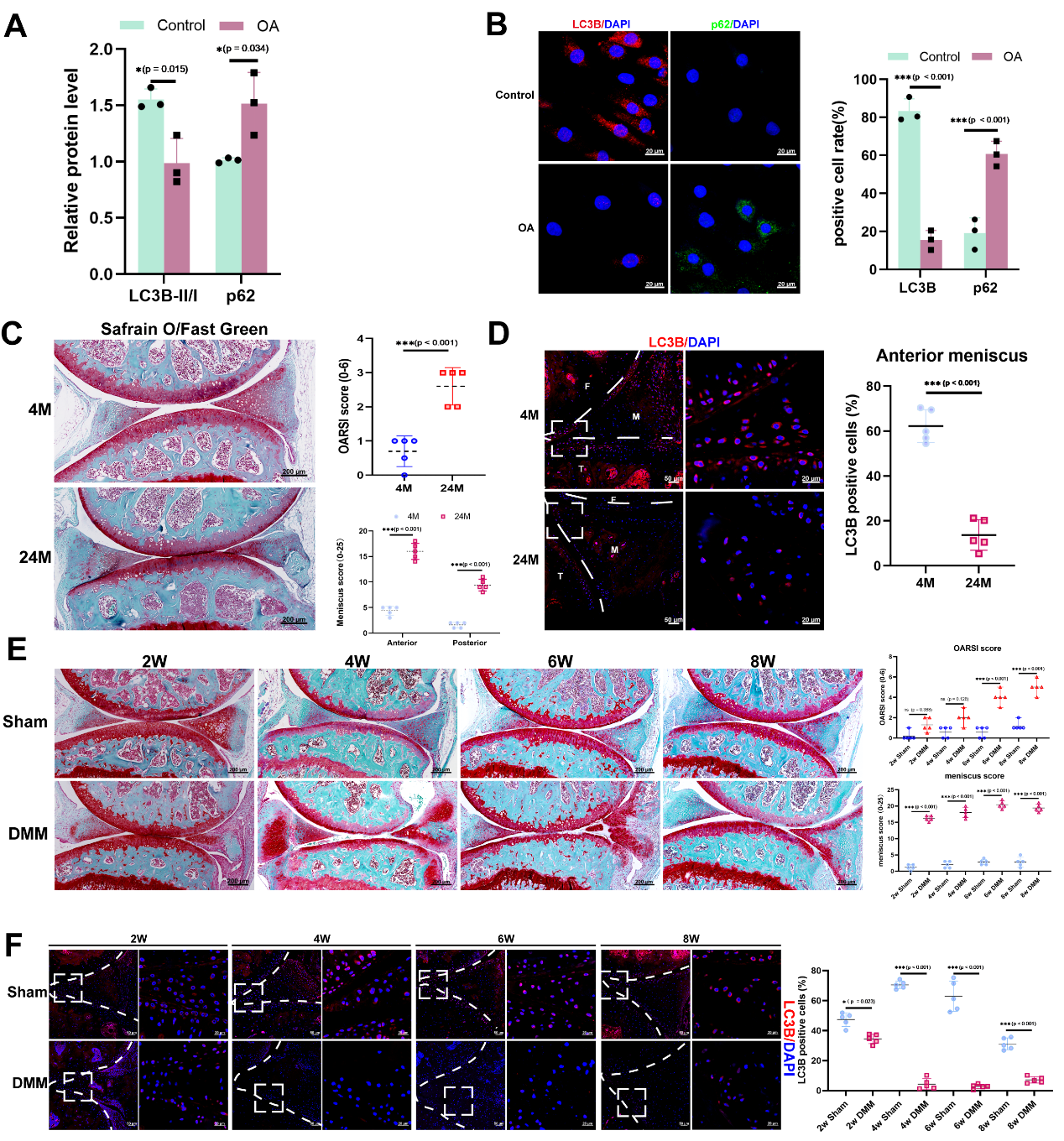


**Figure S2. Impaired autophagy in meniscus cells with OA.**

(A) The quantification analysis of relative protein levels of LC3B-II and p62 n = 3 per group.

(B) The representative confocal IF images of LC3B (red) and p62 (green) and the quantification analysis of the percentage of LC3B and p62 between meniscus cells with and without OA n = 3 per group.

(C) The representative SOFG images of knee tissue and the quantification analysis of meniscus score and OARSI score between 4-month-age mice and 24-month-age mice n = 5 per group.

(D) The representative IF images of LC3B (red) in meniscus tissues and quantification analysis between 4-month-age mice and 24-month-age mice n = 5 per group.

(E) The representative SOFG images of knee tissue and the quantification analysis of meniscus score and OARSI score between mice with DMM surgery and Sham surgery after 2 weeks, 4 weeks, 6 weeks, 8weeks n = 5 per group.

(F) The representative IF images of LC3B (red) in meniscus tissues and quantification of the percentage of LC3B in meniscus tissues between mice with DMM surgery and Sham surgery after 2 weeks, 4 weeks, 6 weeks, 8weeks n = 5 per group. Dotted lines marked the location of meniscus. F refers to Femoral cartilage, T refers to Tibia, M refers to meniscus. Unpaired student’s t test (A-D) and one-way ANOVA followed with Turkys’ HSD test (E and F) were used for statistical analysis and p values were marked on each bar plots.

**Figure S3.**


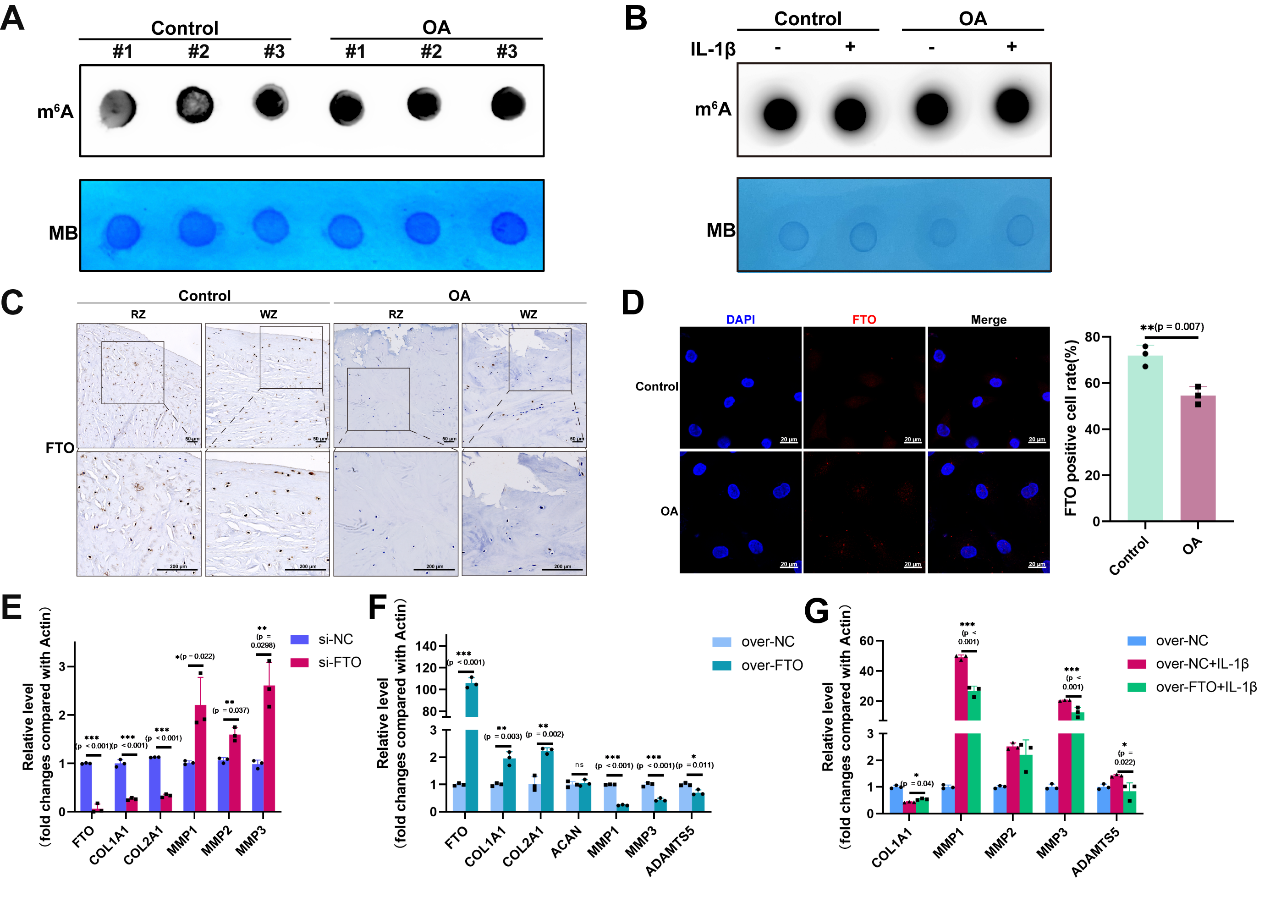


**Figure S3. FTO regulates meniscus degeneration during OA.**

(A) The dot blot assay of m^6^A in meniscus cells from patients with and without OA n = 3 per group.

(B) The dot blot assay of m^6^A in meniscus cells with and without IL-1β stimulation.

(C) The representative IHC staining images of FTO in meniscus tissues from VAS area and AVAS area with and without OA.

(D) The representative confocal images of IF staining of FTO (red) and the quantification of FTO percentage in meniscus cells with and without OA n = 3 per group.

(E) The qRT-PCR analysis of the expression of FTO, COL1A1, COL2A1, MMP1, MMP2, MMP3 in meniscus cells with and without si-FTO transfection n = 3 per group.

(F) The qRT-PCR analysis of the expression of FTO, COL1A1, COL2A1, ACAN, MMP1, MMP3, ADAMTS5 in meniscus cells with and without oe-FTO transfection n = 3 per group.

(G) The quantification analysis of the expression of COL1A1, MMP1, MMP2, MMP3, ADAMTS5 in over-NC, over-NC + IL-1β, over-FTO + IL-1β group n = 3 per group. Unpaired student’s t test (D-F) and one-way ANOVA (G) were used for statistical analysis and p values were marked on each bar plots . RZ refers to Red Zone of meniscus, and WZ refers to White Zone of meniscus.

**Figure S4.**


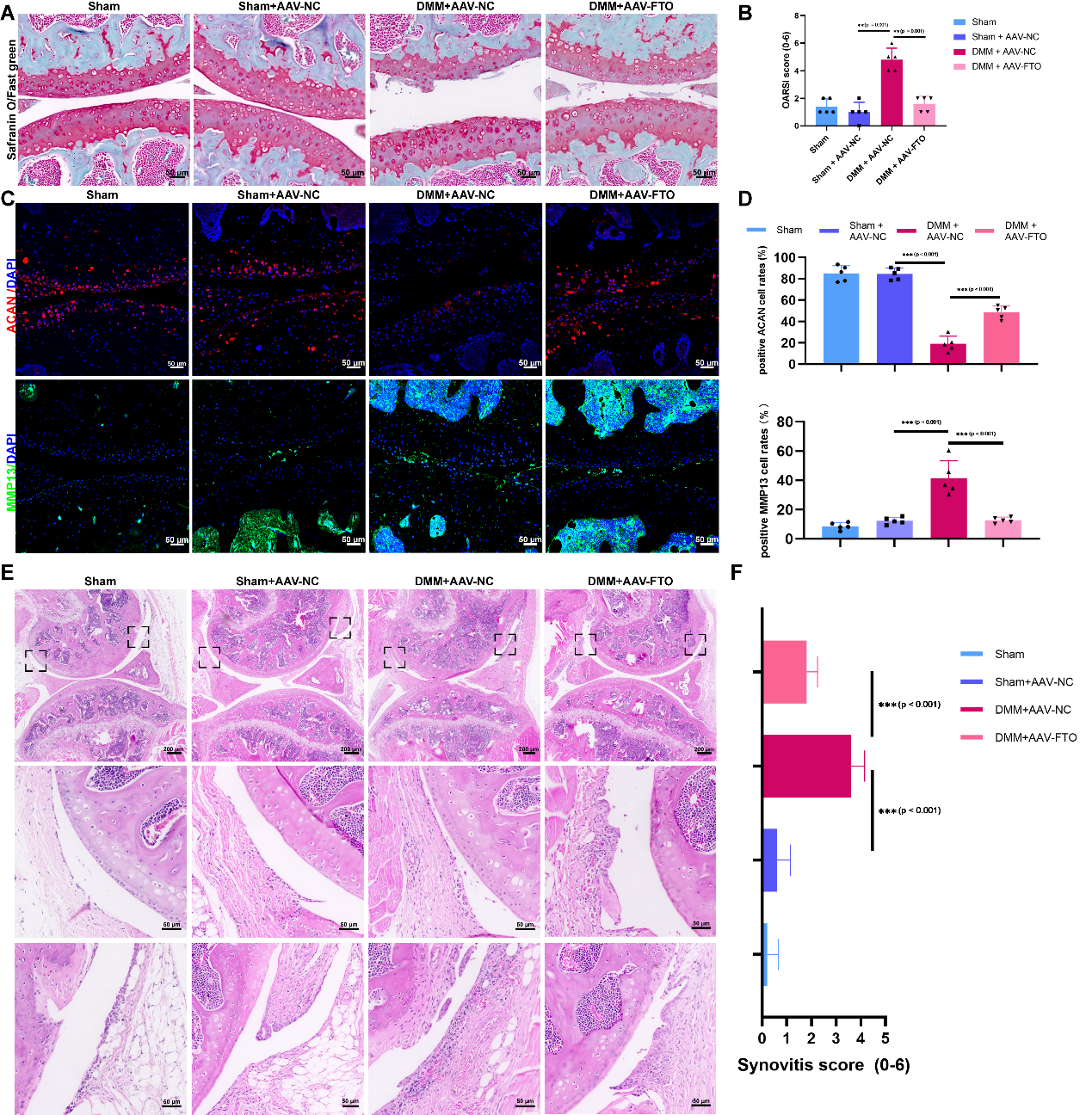


**Figure S4. Intra-articular injection of AAV overexpressing FTO alleviates DMM-induced OA in mice.**

(A-B) The representative SOFG staining and quantification analysis of OARSI grading in Sham, Sham + AAV-NC, DMM + AAV-NC, DMM + AAV-FTO groups n = 5 per group.

(C-D) The representative IF staining of ACAN (red), MMP13 (green) and the quantification analysis of the percentage of ACAN and MMP13 in Sham, Sham + AAV-NC, DMM + AAV-NC, DMM + AAV-FTO groups. n = 5 per group.

(E-F) The representative HE staining of synovium and the quantification analysis of synovitis score Sham, Sham + AAV-NC, DMM + AAV-NC, DMM + AAV-FTO groups. n = 5 per group. In all IF images, dotted lines marked the location of meniscus. F refers to Femoral cartilage, T refers to Tibia, M refers to meniscus. One-way ANOVA followed with Turky’s HSD test (Figure S4B, D, F) were used for statistical analysis and p values were marked on each bar plots.

**Figure S5.**


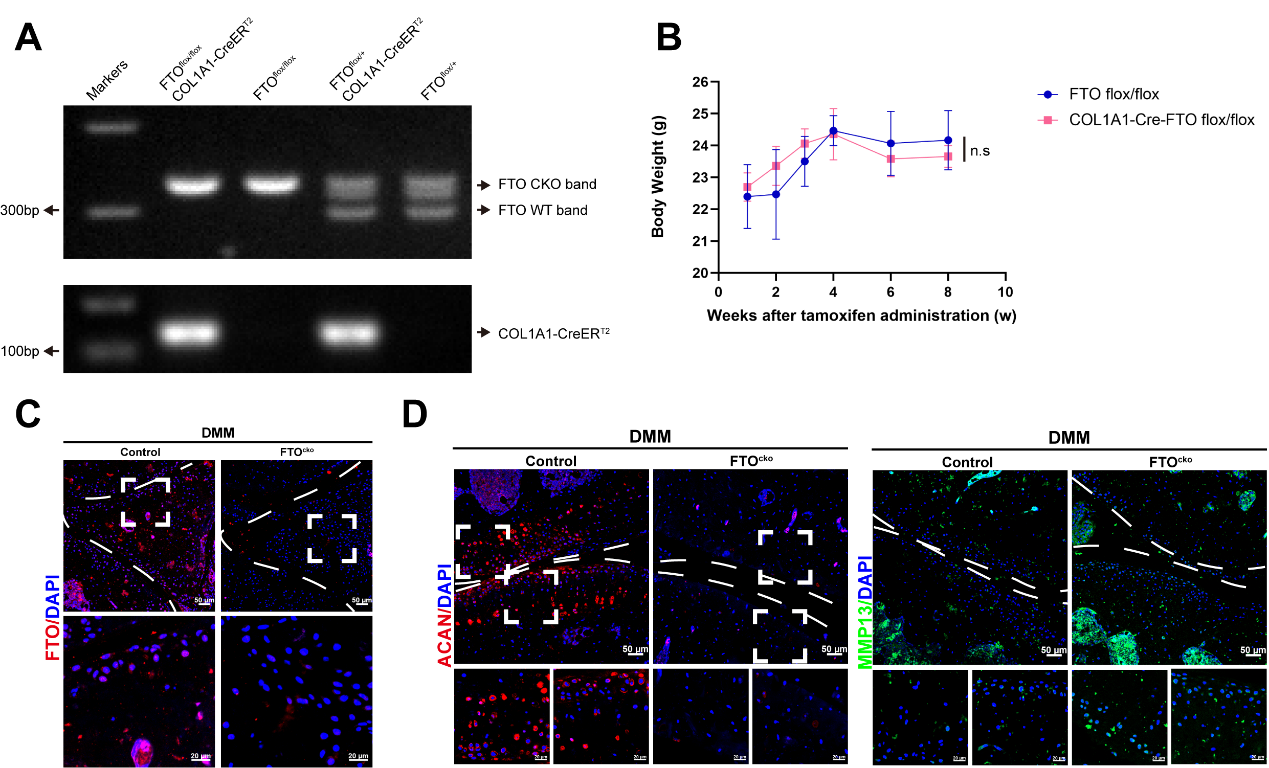


**Figure S5. The identification of FTO^cko^ mice.**

(A) The genotype identification using PCR (KO band 300 bp, WT band 250 bp).

(B) The graph of the weight between control and FTO^cko^ mice each weeks after tamoxifen induction n = 5 per group.

(C) The representative IF staining images of FTO (red) in meniscus tissues from control and FTO^cko^ mice. Dotted lines marked the location of meniscus.

(D) The representative confocal images of IF staining of ACAN (red) and MMP13 (green) and the quantification of ACAN and MMP13 percentage in chondrocyte in control and FTO^cko^ mice.

**Figure S6.**


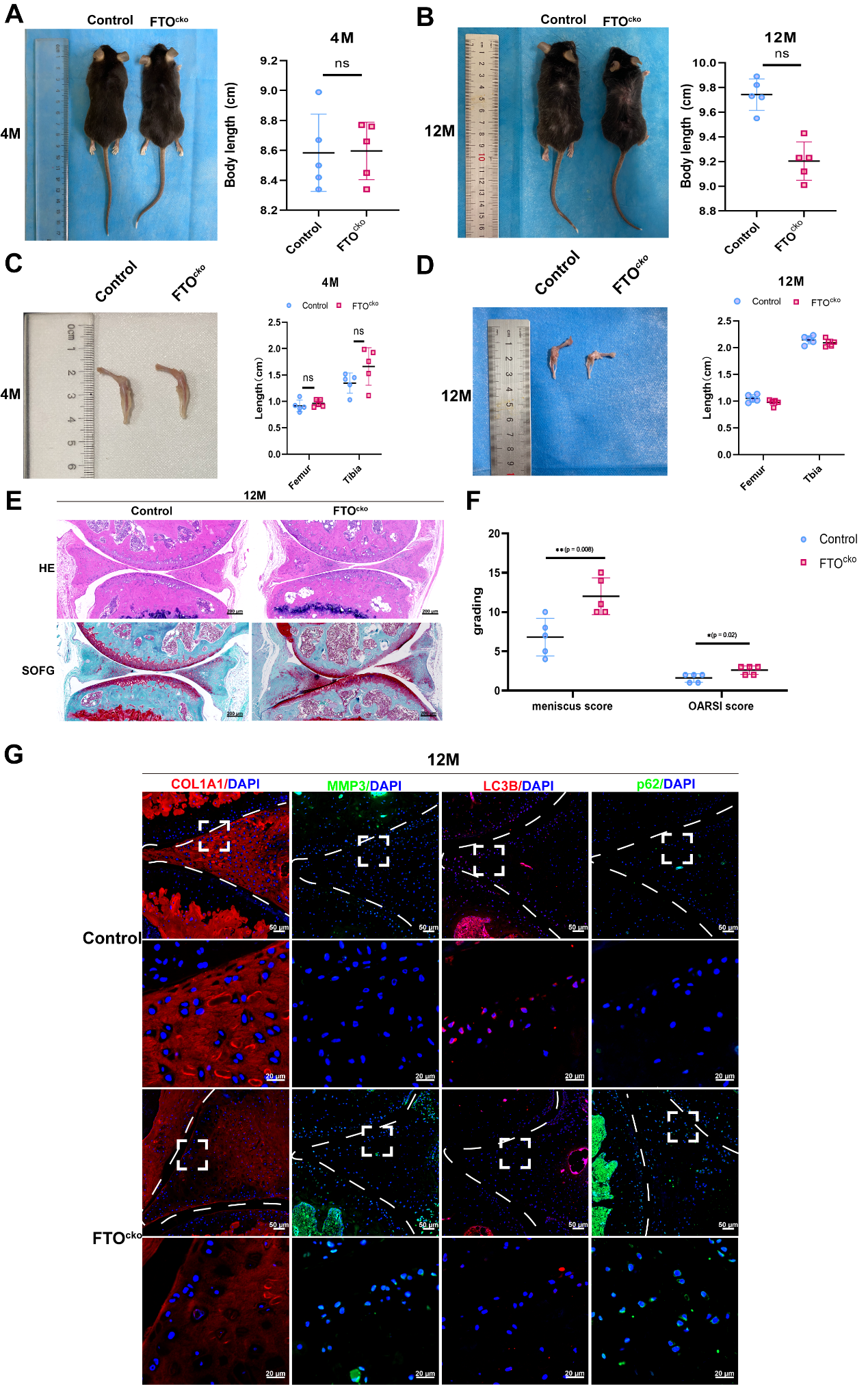


**Figure S6. The OA phenotype of aging models of FTOcko mice.**

(A) The gross morphology and the quantification of body length (cm) between 4-month-age control and FTOcko mice. n = 5 per group.

(B) The gross morphology and the quantification of body length (cm) between 12-month-age control and FTOcko mice. n = 5 per group.

(C) The gross morphology and the quantification of length in lower limb (cm) between 4-month-age control and FTOcko mice. n = 5 per group.

(D) The gross morphology and the quantification of length in lower limb (cm) between 12-month-age control and FTOcko mice. n = 5 per group.

(E) The representative HE and SOFG staining of the knee joint of control and FTO^cko^ mice aged 12 months.

(F) The evaluation of meniscus degeneration and cartilage degeneration in control and FTOcko mice based on meniscus score and OARSI score n = 5 per group.

(G) The representative IF staining of COL1A1, MMP3, LC3B, p62 in the meniscus of control and FTO^cko^ mice. In all IF images, dotted lines marked the location of meniscus. Unpaired student’s t test (Figure S6A-D, F) was used for statistical analysis and p values were marked on each bar plots.

**Figure S7.**


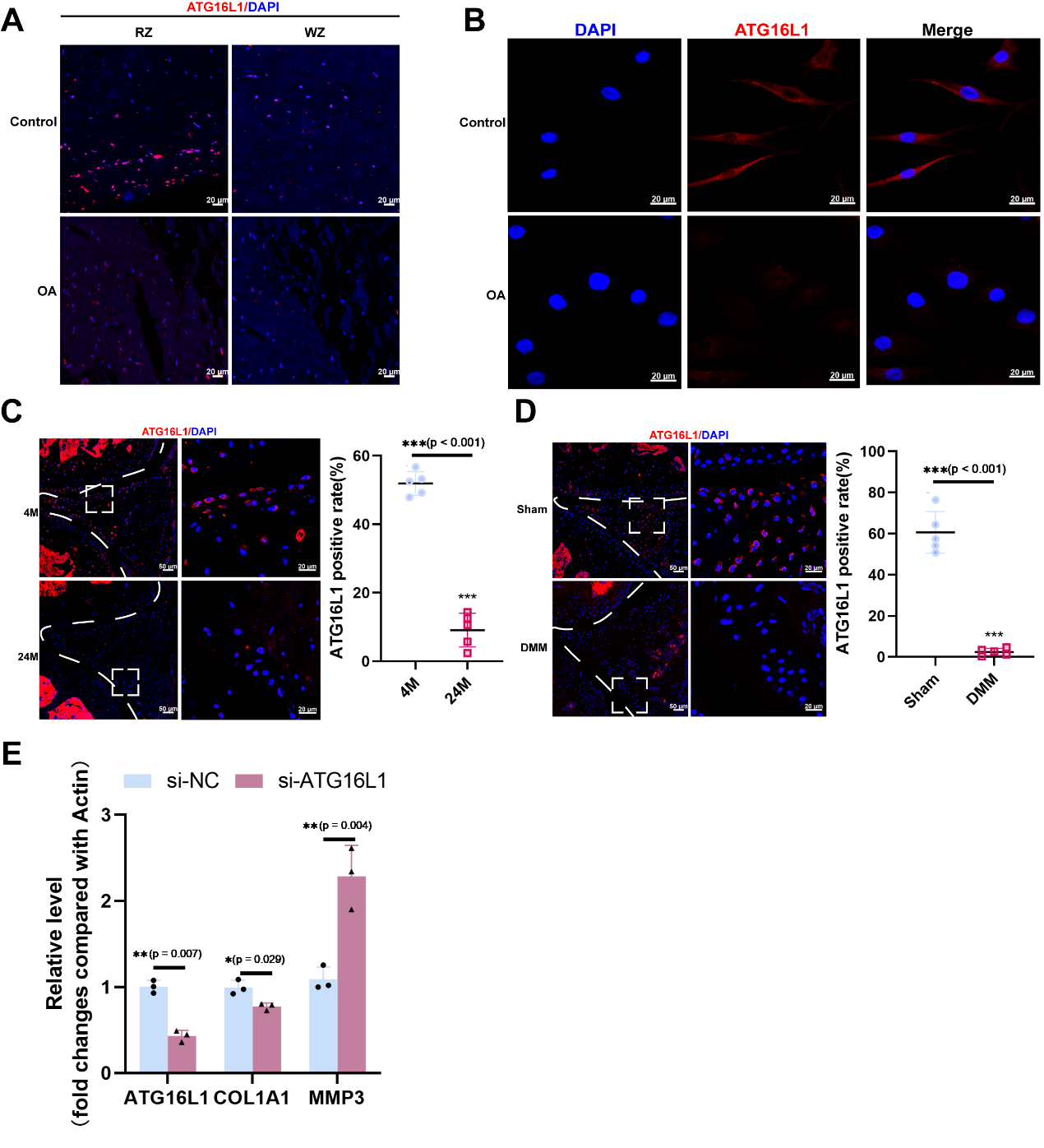


**Figure S7. ATG16L1 regulates meniscus degeneration during OA.**

(A) The representative IF stainings of ATG16L1 (red) of meniscus tissues with and without OA.

(B) The representative confocal images of IF stainings of ATG16L1 (red) of primary meniscus cells with and without OA.

(C) The representative IF stainings and quantification of ATG16L1 in meniscus tissues between 4-month-age mice and 24-month-age mice. n = 5.

(D) The representative IF stainings and quantification of ATG16L1 in meniscus tissues between mice with and without DMM-induced OA models. n = 5.

(E) The qRT-PCR of the expression of ATG16L1, COL1A1, MMP3 in meniscus cells with and without si-ATG16L1. n = 3. In all IF images, dotted lines marked the location of meniscus. Unpaired student’s t test (Figure S7C-E) was used for statistical analysis and p values were marked on each bar plots. RZ refers to Red Zone of meniscus, and WZ refers to White Zone of meniscus.

**Figure S8.**


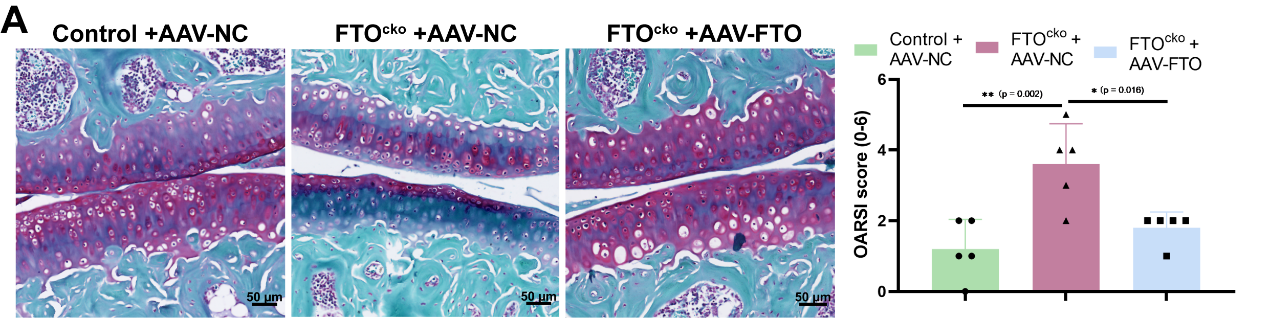


**Figure S8. Intra-articular injection of AAV-FTO alleviates OA in FTO^cko^ mice.**

(A) The representative SOFG staining of control + AAV-NC, FTO^cko^ + AAV-NC, and FTOcko + AAV-FTO groups. And the evaluation of OARSI score between control + AAV-NC, FTO^cko^ + AAV-NC, and FTO^cko^ + AAV-FTO groups n = 5. one-way ANOVA followed with Turky’s HSD test (A) were used for statistical analysis and p values were marked on each bar plots.

**Table S1. Differential detected metabolites between meniscus cells with and without OA**

| **Metabolites** | **FoldChange** | **P-value** |
| --- | --- | --- |
| NADH | 0.575121872 | 0.014113552 |
| cis-Aconitate | 2.024879585 | 0.072590572 |
| Dihydroxyacetone phosphate | 2.075836676 | 0.007365866 |
| Beta-D-Fructose 6-phosphate | 2.380116139 | 0.071149462 |
| D-Glucose 6-phosphate | 2.553656464 | 0.077272737 |
| GMP | 2.528208532 | 0.036201798 |
| Phosphoenolpyruvate | 3.313488591 | 0.162794772 |
| Oxaloacetate | 0.60134192 | 0.274001626 |
| Citrate | 1.576503197 | 0.081163402 |
| Isocitrate | 1.89768807 | 0.123272329 |
|  |  |  |

**Table S2. Information of patients recruited in this study**

| **Patient number** | **Gender** | **Age** | **Kellgren-Lawrence**  **grading scale** |
| --- | --- | --- | --- |
| 1 | M | 20 | 0 |
| 2 | M | 16 | 0 |
| 3 | M | 30 | 1 |
| 4 | F | 26 | 1 |
| 5 | F | 33 | 1 |
| 6 | F | 67 | 4 |
| 7 | F | 68 | 4 |
| 8 | M | 70 | 4 |
| 9 | M | 65 | 4 |
| 10 | F | 72 | 4 |

**Table S3. Primers used for qRT-PCR**

| **Gene** | **Primer sequence (5’-3’)** |
| --- | --- |
| hsa-COL1A1-F | GAGGGCCAAGACGAAGACATC |
| hsa-COL1A1-R | CAGATCACGTCATCGCACAAC |
| hsa-COL2A1-F | TCCTGCCGTTTCGCTG |
| hsa-COL2A1-R | CATTATACCTCTGCCCATCCTG |
| hsa-COL3A1-F | GCCAAATATGTGTCTGTGACTCA |
| hsa-COL3A1-R | GGGCGAGTAGGAGCAGTTG |
| hsa-COL6A1-F | TCTGCATAGACAAGAAGTGTCCA |
| hsa-COL6A1-R | GGTGTCAAAGTTGTGGCTGC |
| hsa-ACAN-F | GGGACCTGCAAGGAGACAGAG |
| hsa-ACAN-R | TCAATCTCACACAGGTCCCCTTC |
| hsa-MMP1-F | CTCTGGAGTAATGTCACACCTCT |
| hsa-MMP1-R | TGTTGGTCCACCTTTCATCTTC |
| hsa-MMP3-F | CTGGACTCCGACACTCTGGA |
| hsa-MMP3-R | CAGGAAAGGTTCTGAAGTGACC |
| hsa-MMP13-F | TCGGCCACTCCTTAGGTCTT |
| hsa-MMP13-R | AAGTGGCTTTTGCCGGTGTA |
| hsa-ADAMTS5-F | GGGCACTGGCTACTATGTGG |
| hsa-ADAMTS5-R | CGTCACAGCCAGTTCTCACA |
| hsa-ADAMTS4-F | GTCCCATGTGCAACGTCAAG |
| hsa-ADAMTS4-R | ATGCGGCCATCTTGTCATCT |
| hsa-COL10A1-F | CATAAAAGGCCCACTACCCAAC |
| hsa-COL10A1-R | ACCTTGCTCTCCTCTTACTGC |
| hsa-ACTB-F | CATGTACGTTGCTATCCAGGC |
| hsa-ACTB-R | CTCCTTAATGTCACGCACGAT |
| hsa-METLL3-F | TTGTCTCCAACCTTCCGTAGT |
| hsa-METLL3-R | CCAGATCAGAGAGGTGGTGTAG |
| hsa-METLL14-F | GAGTGTGTTTACGAAAATGGGGT |
| hsa-METLL14-R | CCGTCTGTGCTACGCTTCA |
| hsa-WTAP-F | ACTGGCCTAAGAGAGTCTGAAG |
| hsa-WTAP-R | GTTGCTAGTCGCATTACAAGGA |
| hsa_ALKBH5_F | CGGCGAAGGCTACACTTACG |
| hsa_ALKBH5_R | CCACCAGCTTTTGGATCACCA |
| hsa_FTO_F | ACTTGGCTCCCTTATCTGACC |
| hsa_FTO_R | TGTGCAGTGTGAGAAAGGCTT |
| hsa-ULK1-F | GGCAAGTTCGAGTTCTCCCG |
| hsa-ULK1-R | CGACCTCCAAATCGTGCTTCT |
| hsa-ATG3-F | GACCCCGGTCCTCAAGGAA |
| hsa-ATG3-R | TGTAGCCCATTGCCATGTTGG |
| hsa-ATG4B-F | GGTGTGGACAGATGATCTTTGC |
| hsa-ATG4B-R | CCAACTCCCATTTGCGCTATC |
| hsa-ATG4D-F | CCAGCCCACTGTGGATGTC |
| hsa-ATG4D-R | AAGCCCACGGTACAGCTTG |
| hsa-ATG5-F | AAAGATGTGCTTCGAGATGTGT |
| hsa-ATG5-R | CACTTTGTCAGTTACCAACGTCA |
| hsa-ATG7-F | ATGATCCCTGTAACTTAGCCCA |
| hsa-ATG7-R | CACGGAAGCAAACAACTTCAAC |
| hsa-ATG10-F | AGACCATCAAAGGACTGTTCTGA |
| hsa-ATG10-R | GGGTAGATGCTCCTAGATGTGAC |
| hsa-ATG12-F | CTGCTGGCGACACCAAGAAA |
| hsa-ATG12-R | CGTGTTCGCTCTACTGCCC |
| hsa-ATG13-F | TTGCTATAACTAGGGTGACACCA |
| hsa-ATG13-R | CCCAACACGAACTGTCTGGA |
| hsa-ATG14-F | TTCAGAGGCATAATCGCAAACT |
| hsa-ATG14-R | CCAGACGCTCATAATGACTTCTT |
| hsa-ATG16L1-F | TCTGGGACATTCGATCAGAGAG |
| hsa-ATG16L1-R | CCTTTCTGGGTTTAAGTCCAGG |
| hsa_ATG16L1_m^6^A site_F | GTGTTCTCTGTGCTTTCTCTCGT |
| hsa_ATG16L1_m^6^A site_R | CCAATGCTTAAAGTGAGTTCACC |

**Table S4. Primers used for qRT-PCR**

| **REAGENT or RESOURCE** | **SOURCE** | **IDENTIFIER** |
| --- | --- | --- |
| **Antibodies** | | |
| Rabbit anti-COL1A1 | abcam (WB)  abclonal (IF) | ab138492 (WB),  A1352 (IF) |
| Rabbit anti-LC3B | Cell Signaling Technology | 43566 |
| Rabbit anti-p62 | Proteintech | 18420-1-AP |
| Rabbit anti-MMP3 | abcam | ab52915 |
| Rabbit anti-ACAN | Proteintech | 13880-1-AP |
| Rabbit anti-m^6^A | Synaptic Systems | 202003 |
| Rabbit anti-MMP13 | Abcam | ab315267 |
| Rabbit anti-FTO | Abcam (WB)  Proteintech (IF) | ab126605 (WB)  27226-1-AP (IF) |
| Rabbit anti-ATG16L1 | Abcam | ab187671 |
| Rabbit anti-YTHDF1 | Proteintech | 17479-1-AP |
| Rabbit anti-YTHDF2 | Proteintech | 24744-1-AP |
| HRP-conjugated Beta Actin Monoclonal antibody | Proteintech | HRP-66009 |
| **Chemical, peptides, and recombinant proteins** |  |  |
| 5 × sample loading buffer (Thermo Fisher) | CWBIO | Cat#CW0052S |
| DAPI | Solarbio | S2110 |
| Dulbecco's Modified Eagle Medium：F12 = 1:1 | Gibco | C11330500B |
| Fetal bovine serum | Vivacell | C04001-500 |
| TRIzol reagent | Invitrogen | Cat#15596018 |
| PBS | Solarbio | Cat#P1031 |
| **Critical commercial assays** |  |  |
| BCA protein assay kit | Thermo Fisher | Cat#23227 |
| Seahorse XF Cell Mito Stress Test Kit | Agilent | 103015-100 |
| Seahorse XF Cell Glycolysis Stress Test Kit | Agilent | 103020-100 |
| Magna MeRIP m^6^A Kit | Millipore | Cat# 17-10499 |
| SYBR Green qPCR Master Mix | AG | AG11701 |
| **Experimental models: organisms/strains** |  |  |
| C57BL/6 mouse | Gempharmatech | N/A |
| COL1A1-CreE^RT2^ mouse | The Jackson Laboratory | N/A |
| FTO^flox/flox^ mouse | Modelorg | N/A |
| **Software and algorithms** |  |  |
| GraphPad Prism | GraphPad | https://www.graphpad.com/  scientific-software/prism/ |
